# Community Views of Determinants of Men’s Wellbeing in Guatemala: A Study Using Fuzzy Cognitive Mapping

**DOI:** 10.1177/2752535X241312378

**Published:** 2025-01-15

**Authors:** Katherine W. Pizarro, Anne M. Chomat, Diego P. Quieju, Bernardo Y. López, Iván Sarmiento, Nicholas LeBel, Chloe Mancini, Neil Andersson, Danielle Groleau, Anne Cockcroft

**Affiliations:** 1 Division of Social and Transcultural Psychiatry, Faculty of Medicine and Health Sciences, 5620 McGill University, Montreal, Canada; 2 Participatory Research at McGill (CIET-PRAM), Department of Family Medicine, Faculty of Medicine and Health Sciences, 5620 McGill University, Montreal, Canada; 3 Centro de Investigación de Enfermedades Tropicales, Inc, Quetzaltenango, Guatemala; 4 Proyecto Buena Semilla, Quetzaltenango, Guatemala; 5 Uxiljuj Batz’, Santiago Atitlán, Guatemala; 6 Escuela de Medicina y Ciencias de la Salud, Universidad del Rosario, Bogotá, Colombia; 7 Faculty of Science, University of Ottawa, Ottawa, Canada; 8 5622 University of Montreal, Montreal, Canada; 9 Centro de Investigación de Enfermedades Tropicales (CIET), Universidad Autónoma de Guerrero, Acapulco, Mexico; 10 Culture and Mental Health Research Unit, Jewish General Hospital, Montreal, Canada

**Keywords:** mental health, public health, qualitative research, psychiatry

## Abstract

**Background:**

In post-conflict Guatemala, Indigenous men’s psychological distress has been linked to violence exposure, disrupted social support systems, and structural inequities.

**Purpose:**

We aimed to document how communities themselves understand men’s wellbeing and the factors that influence men’s wellbeing.

**Research design and study sample:**

Fuzzy Cognitive Mapping with 20 stakeholder groups in Santiago Atitlán and Cuilco, Guatemala defined men’s wellbeing in local terms and identified the influences community groups understood to promote and detract from men’s wellbeing. Participants mapped pathways through which influences affected wellbeing and weighted their relative perceived strength.

**Analysis:**

The researchers used thematic analysis to summarise influences into 43 factors and used fuzzy transitive closure to calculate their net causal influence for each set of stakeholders. We compared perspectives of groups of adult men, adult women, and practitioners of Mayan medicine in Santiago Atitlán, with a primarily Indigenous population, to groups in Cuilco, with a primarily non-Indigenous population. We also compared perspectives across age groups in Santiago Atitlán.

**Results:**

Across regions, maps highlighted the importance of family and social relations, emotional distress, substance use and physical health for men’s wellbeing. Basic resource insecurity and unemployment were top risk factors for men’s wellbeing in maps from Cuilco but had both risk and protective influences on men’s wellbeing in maps from Santiago Atitlán.

**Conclusions:**

Findings challenge the focus on scale-up of individual biomedical interventions as the best strategy to reduce the burden of emotional distress in Guatemala and raise questions about standard development approaches that emphasize income generation and educational attainment above cultural continuity and social harmony.

## Introduction

Concern about the number of people who require mental health treatment but do not receive it^1^(p. 463) has led the World Health Organization to create the Mental Health Gap Action Programme (mhGAP) to support scaling up evidence-based mental health services around the world.^2^ This focus on evidence-based mental health care has fostered an individualized, biomedical view of mental health in policy and practice, downplaying the social, political and structural determinants of psychological distress.^3,4^ Researchers have called for more research on psychosocial interventions, including those that address the social and structural conditions leading to psychological distress and those that foster Indigenous forms of healing.^5,6^ Greater understanding of Indigenous perspectives can inform the design of more relevant psychosocial interventions and ensure that the indicators and tools for evaluation align with their values and priorities.^7^

In Guatemala, much attention has been given to the prolonged period of state-sponsored violence, that ended in 1996 after 200,000 primarily Maya citizens were killed, as a cause of ongoing psychosocial distress. A nationally representative survey found an association between previous experience of violence and post-traumatic stress disorder (Adjusted odds ratio (AOR) = 52.7 for Indigenous participants), with higher odds among those who experienced violence during the period of armed conflict.^8^ Qualitative and ethnographic research in Guatemala has identified additional social and structural determinants of distress in the post-war period, including economic insecurity and difficulty meeting basic needs,^9,10^ and social fragmentation and distrust.^11,12^ Wellbeing of Indigenous communities in Guatemala is affected by ongoing threats to their cultural and religious practices, widespread discrimination, violence, and a health system that discourages traditional practices and does not accommodate non-Spanish speakers.^13–16^ While a gender lens has usefully been applied to research on Indigenous women’s health, research focused on the mental health and wellbeing of Indigenous men is scarce. Despite research highlighting high rates of alcohol use disorders, domestic violence,^17,18^ and suicidal ideation^19^ among men in Guatemala, to our knowledge, no research has focused on the specific determinants of men’s psychosocial wellbeing or distress.

There exists limited research on how Indigenous communities in Guatemala understand psychosocial wellbeing or the factors that promote or detract from wellbeing. Systematizing local knowledge about factors that may protect Indigenous men in Guatemala from psychosocial distress and associated harmful behaviors is an important step in promoting their health and that of their families. We chose wellbeing as the central focus in our research to shift away from deficit-based research with Indigenous communities toward a holistic, strengths-based understanding of Indigenous people’s psychosocial needs.^5,20^

Fuzzy Cognitive Mapping (FCM)^21^ is a useful tool to engage stakeholders in systematically identifying influences on an outcome, while supporting collective learning and decision making.^22^ It engages participants in identifying all influences that they understand to contribute to an outcome, making perceived causal pathways explicit and encouraging reflection on how multiple influences are inter-related.^23^ Since its introduction in the early eighties, FCM has attempted to open space for non-Western views of causal understanding.^24^ Although it is not an Indigenous methodology, FCM has been useful in facilitating communication between researchers and Indigenous communities in Canada,^25–27^ Mexico,^28,29^ and six other countries.^30^ The technique has proven robust and flexible across different cultural and educational backgrounds, facilitating intercultural dialogue.^31^ This aligns with our goal of bringing findings into conversations on the scale-up of mental health services, as well our plan to use the findings to develop survey instruments to evaluate a strengths-based, community-led approach to promoting wellbeing.

In this study, we used FCM to explore and systematize the knowledge of communities in Guatemala about the meaning of men’s wellbeing and factors that influence it. We sought to compare perspectives from two geographic regions—one with a primarily Indigenous population known for its strong connection to Indigenous cultural practices and another where most of the population identifies as non-Indigenous. We also sought to compare perspectives of adult men, adult women and practitioners of traditional medicine, as well as of groups of different ages (young adults, older adults). These comparisons contrasted perspectives according to lived experiences and engagement in Indigenous cultural practices. We anticipated that these experiences would vary across regions, levels of cultural continuity, and among generations, particularly between those who lived through the armed conflict and those who grew up in the post-war period.

## Methods

### Study Design and Public Involvement

Our research was part of a pilot intervention that engaged marginalized communities in Guatemala in planning strategies to promote wellbeing. FCM facilitated public involvement in defining wellbeing and prioritizing actions to promote it. It was the first step in engaging key stakeholders in the co-design process. Stakeholder maps formed the evidence base for community dialogues in which participants discussed the key influences on wellbeing and planned actions to improve it. In addition to review and approval by institutional research ethics committees, we met with Huehuetenango and Sololá Department of Health officials and community committees to seek input and approval for this study.

### Setting

The research took place in two municipalities in Western Guatemala: Santiago Atitlan, Sololá, and Cuilco, Huehuetenango. Cuilco’s population is almost entirely rural, while Santiago Atitlán contains a small urban center (total population <50,000).^32^ Both municipalities are located over 100 km from the capital of Guatemala. The population of Santiago Atitlán is 97% Indigenous, primarily from the *Tz’utujil* Maya ethnic group. The *Tz’utujil* people have retained a strong connection to Indigenous cultural practices compared with other Indigenous groups in Guatemala, due to their remote location and relative lack of colonial interest in the region.^33^ Nevertheless, sociocultural change is accelerating in recent years, due to the dynamics of globalization.^33^ In Cuilco, only 20% of the population identifies as Indigenous, primarily from the *Mam* Maya ethnic group, while 80% identifies as *ladino*.^32^ While the term *ladino* is used to imply non-Indigenous status, *ladino* populations include individuals of mixed Indigenous and European ancestry, as well as individuals with Indigenous ancestry who have abandoned Indigenous identity through a shift in language, dress, and cultural practices.^34^

## Participants

Two communities in each municipality took part in the study (total participating communities = 4). Both communities in Cuilco were rural, each with populations less than 2,000. One community in Santiago Atitlán was rural (population 3,395) and one was urban (population 8,269). With the assistance of local health post staff and community authorities, local facilitators identified and invited a convenience sample of participants to attend mapping workshops with separate stakeholder groups, aiming for 7-10 participants per session. Participants were primarily identified based on their affiliation with the health post (e.g. known *abuelas comadronas*, women who had received prenatal care) or, in the case of Santiago Atitlán, through the local research team member’s familiarity with the community. Recruitment took place in person. In Santiago Atitlán, facilitators convened separate groups of young men, young women, adult men, adult women, older men, older women, and *Terapeutas Mayas* (traditional Indigenous practitioners) in each community (14 groups total). In Cuilco, due to greater recruitment challenges, facilitators convened one group each of adult men, adult women and *Terapeutas tradicionales* per community (6 groups total). *Terapeutas Mayas* (as they are known in Santiago Atitlán) and *Terapeutas tradicionales* (as they are known in Cuilco) included *abuelas comadronas* (traditional midwives), *ajq’ij* (Mayan spiritual guides), and *terapeutas* (therapists) who use ancestral knowledge to treat common illnesses and/or accompany women in the period surrounding childbirth in their communities. Inclusion criteria included: (a) self-identifying as a member of a particular group (e.g. young women), (b) providing informed consent, (c) being over 18 years of age or an emancipated minor, (d) residing in the study community, and (e) speaking Spanish in Cuilco or Tz’utujil in Santiago Atitlán. We did not use strict age cutoffs for group participation but rather relied on self-identification as a young adult, adult, or older adult to determine group participation. This allowed participants to make decisions based on life experiences, such as becoming a parent or grandparent, and local views of what each category means.

### Data Collection

We used a mapping protocol that emphasizes participation of marginalized voices, which has been used with Indigenous groups in Mexico.^21,35^ Our team pilot tested the protocol with local health workers prior to data collection and verified the relevance of the central concept through informal conversations with community members in Cuilco and Santiago Atitlán. To ensure participants were considering “wellbeing” as understood in their context, each mapping session started by asking participants what wellbeing meant for men *in their community*. Data collection took place from July to October of 2018. Most maps were created during a single meeting that lasted 2.5 to 4 hours, with some requiring completion at a second meeting. The mapping sessions took place at local health posts, in local community buildings, or at the facilitator’s home. Local male facilitators (DPQ, BYL) with post-secondary education facilitated the mapping sessions in the local language (Spanish in Cuilco, *Tz’utujil* and Spanish in Santiago Atitlán), alongside a local female research assistant for the women's groups. The female first author was present for some of the mapping sessions with women’s groups and the male study coordinator was present for some sessions with men’s groups. They are trained and experienced group facilitators and did not play an active role in the sessions; we do not believe their presence disturbed the group dynamics. Some participants had children present during the sessions.

Facilitators first explained the purpose of the activity and asked participants for oral informed consent. The activity was introduced as part of a project that aimed to engage local community groups in promoting wellbeing in their communities. Oral consent was appropriate given the minimal risk involved, local norms for indicating consent, and low literacy rates. Facilitators approached participants individually to collect basic sociodemographic data, including age, marital status, occupation, highest education level, languages spoken and having children (yes/no).

To initiate the mapping session, facilitators asked participants, as a group, to define what wellbeing meant for men in their community. This allowed us to document the meaning of the concept for each group and center the discussion on participant perspectives.^35^ The facilitator then asked participants to name the influences on men’s wellbeing in their community; the facilitator wrote these on sticky notes and placed them on a poster until participants felt the list was exhaustive. The facilitator then asked participants to describe how each concept affected wellbeing, either directly or indirectly through another concept or concepts. The facilitator drew arrows on the poster to illustrate each of these relationships, as explained by participants. Finally, the facilitator asked participants to rate the strength of each connection on a scale of one to five, with five representing the strongest influence and one representing the weakest, and with positive signs indicating positive (promotive) relationships and negative signs indicating negative (inhibitory) relationships. Individual participants contributed concepts related to wellbeing. The group made decisions about links between concepts and weights allotted to links by consensus. Some decisions required debate but none of the facilitators reported any instances of conflict or need of voting. The outputs of the activity were twofold: definitions of men’s wellbeing, and the maps themselves.

### Analysis

#### Definition of Wellbeing

The first author used an inductive approach to thematic content analysis to categorize the concepts included in participant definitions of men’s wellbeing.

#### Fuzzy Cognitive Maps

The maps generated by stakeholders are a type of directed graph, whose properties can be analyzed using the tools of graph theory.^36^ To facilitate comparison of maps, we first conducted an inductive thematic content analysis of concepts included in the original maps. The first author, the study coordinator and the two local facilitators met over several days to discuss each concept in detail and merge into factors concepts that had identical or similar meanings but were expressed differently across maps. Through an iterative process of thematic analysis, concepts were further grouped until we reached consensus on a final set of 43 factors linked to the outcome of men’s wellbeing. Each of the original maps was transformed into a factor map, replacing original concepts with corresponding factor names.

In order to compare the maximum causal influence of each factor on wellbeing across maps, we calculated fuzzy transitive closure (TC)^29^ on each map using CIETmap open source software, which provides a user-friendly interface with R.^37^ TC calculates the influence of each factor on all other factors in a directed graph by identifying all pathways, both direct and indirect, between each set of factors. Each pathway is assigned the weight of the weakest connection along the pathway, and the value of the strongest (highest value) pathway becomes the final value assigned to the relationship between a set of factors. Positive and negative pathways are calculated separately and summed, so if a given map includes a pathway from (A) to (B) with a weight of +5 and another pathway from (A) to (B) with a weight of −5, the net causal influence of (A) on (B) will be 0. This analysis yielded the net causal influence of each factor on wellbeing for each map, which we normalized on a scale from −1 to 1. Finally, we calculated the combined net causal influence of each factor on wellbeing within each stakeholder group by averaging the TC values across all maps within a given stakeholder group. We refer to these as stakeholder maps. Table 1 displays the number of groups and individual participants included in the analysis of each stakeholder map. Supplemental Table 1 provides a full list of factors and their descriptions.

**Table 1.** Number of Groups and Number of Individuals Participating in FCM by Community and Demographic Group.

Region	Demographic group	# Of groups (# of individual participants)
Cuilco	Adult men	2 (27)
	Adult women	2 (22)
	*Terapeutas tradicionales*	2 (9)
Santiago Atitlán	Young men	2 (9)
	Adult men	2 (24)
	Elder men	2 (8)
	Young women	2 (8)
	Adult women	2 (9)
	Elder women	2 (11)
	*Terapeutas Mayas*	2 (7)
Total		20 (134)

#### Stakeholder Group Comparisons

We compared stakeholder maps from Santiago Atitlán (where most of the population identifies as Indigenous) with maps from Cuilco (where a minority of the population identifies as Indigenous) within each of the stakeholder groups—adult men, adult women and *Terapeutas Mayas/tradicionales*. We also compared stakeholder maps for adult men, adult women, and *Terapeutas Mayas/tradicionales* within each region, and maps of different age groups in Santiago Atitlán (young adults, adults, and older adults).

### Reflexivity

The external researchers planned the research, including deciding which communities to include, in close collaboration with colleagues from the *Instituto de Salud Incluyente (ISIS),* a Guatemala-based institute working to transform the local health system to address the upstream determinants of health and promote health equity, and *Proyecto Buena Semilla,* a Guatemala-based collective that aims to support self-determination and community voices in promoting the wellbeing of marginalized groups in Guatemala. The long-standing relationships between *ISIS, Proyecto Buena Semilla* and the communities made possible the work in these communities.

Group facilitators, who were also involved in analysis, were young men with higher education in social work, and had previous experience with health programming. One identifies as Maya *Tz’utujil*, lives in one of the participating communities, leads a local weaving cooperative, and participates on community committees and in community organizing. The other resides outside the communities where he facilitated mapping workshops. The first author is a white female from the United States who was undertaking a PhD at a Canadian university, with academic training in Indigenous mental health research and community-based mental health interventions. The local study coordinator, who contributed to data analysis, is a Guatemalan medical doctor with many years of experience leading public health, development, and humanitarian aid projects in Guatemala and internationally. Our diverse experiences and worldviews contributed to deliberation around the categorization of concepts included in the maps. We sought a balance between representing local understandings of how concepts fit together and using category names that aligned with Western understandings of health, illness, and their social determinants.

## Results

### Participant Characteristics

Table 2 shows demographic data for the groups of adult men, adult women and *terapeutas Mayas/tradicionales*. All participants in Santiago Atitlán spoke an Indigenous language, but only a minority of participants in Cuilco. In Santiago Atitlán, where secondary analyses included comparisons by age, the average age of participants was 23 for young men, 25 for young women, 70 for older men and 64 for older women. More young adults were single (16/17), without children (16/17), and had at least primary schooling (16/17). No older participants had primary schooling.

**Table 2.** Demographic Characteristics of Participants in Primary Stakeholder Groups.

	Adult women		Adult men		*Terapeutas Mayas/tradicionales* ^a^	
	Santiago Atitlán (*n* = 9)	Cuilco (*n* = 22)	Santiago Atitlán (*n* = 24)	Cuilco (*n* = 27)	Santiago Atitlán (*n* = 7)	Cuilco (*n* = 9)
Average age (range)	34 (26-65)	36 (23-51)	41 (26-55)	39 (25-64)	55 (37-65)	55 (43-84)
Proportion with at least primary schooling	4/9	3/22	14/24	12/27	1/6	0/9
Proportion who spoke an indigenous language	9/9	4/22	24/24	2/22	7/7	3/9
Proportion married or living with a partner	9/9	22/22	23/24	23/27	7/7	9/9
Proportion with children	9/9	22/22	23/24	26/27	7/7	9/9

^a^All *terapeutas* in Santiago Atitlán were women; 8/9 *terapeutas* in Cuilco were women.

### Definition of Wellbeing

Participant definitions of men’s wellbeing from different stakeholder groups included 6 to 27 concepts. All groups included concepts pertaining to family and social relations or personal characteristics that would impact such relations, such as having family support, taking care of family, getting along with neighbors, and being respectful and collaborative. Most groups also included concepts related to having a job, hard work and responsibility; emotional wellbeing and positive thoughts; and physical health and nutrition. Most groups in Cuilco, but only one group in Santiago Atitlán, included concepts related to sports and recreation. Many groups in Santiago Atitlán included concepts related to receiving or following advice from elders, and spirituality or religion, but these concepts rarely featured in Cuilco.

### Structure of Maps

**Figure 1.**
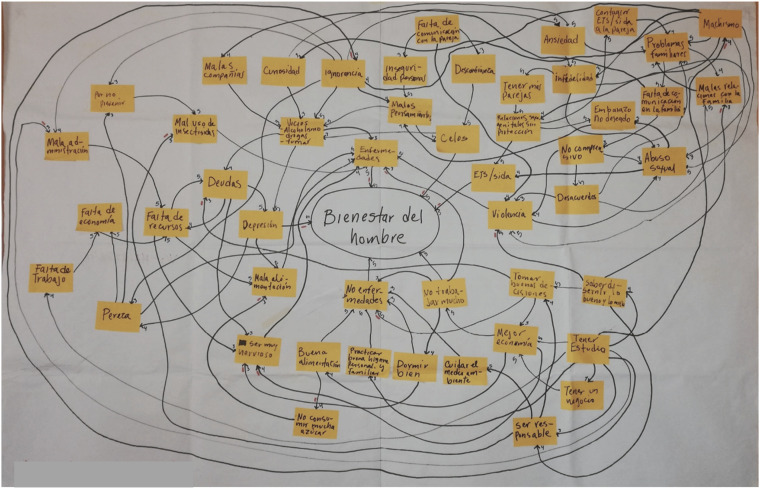
Example of a fuzzy cognitive map created by adult men in Cuilco.

Original maps of men’s wellbeing contained 28 to 64 concepts and 65 to 146 connections. See Figure 1 for an example of an original map created by adult men in Cuilco. Most factors, across all maps, referred to individual-level characteristics or behaviors—such as low self-esteem, personal characteristics that negatively affect social harmony, and substance use, or family-level influences—such as family separation and neglect, infidelity and domestic violence. A few factors implied social or structural influences, such as harmful gender norms and unemployment.

### Regional Comparisons

#### Adult Men

In both Cuilco and Santiago Atitlán, adult men ranked poor physical health, emotional distress, substance use, and factors related to family and social relations (infidelity; personal characteristics that negatively affect social harmony; lack of affectionate, trusting, supportive family relationships) among the most important factors threatening men’s wellbeing (see Table 3).

**Table 3.** Ranking of Factors According to Net Causal Influence on Men’s Wellbeing in Maps From Adult Men, Adult Women and Terapeutas Mayas/Tradicionales in the Two Regions.

	Adult men		Adult women		*Terapeutas Mayas/tradicionales*	
Factor	Santiago Atitlán	Cuilco	Santiago Atitlán	Cuilco	Santiago Atitlán	Cuilco
Poor physical health	1 (−0.95)	4 (−0.93)	1 (−0.60)	2 (−0.70)	1 (−1.00)	1 (−1.00)
Emotional distress	2 (−0.60)	1 (−1.00)	2 (−0.50)	2 (−0.70)	1 (−1.00)	11 (−0.98)
Substance use	2 (−0.60)	1 (−1.00)	2 (−0.50)	2 (−0.70)	1 (−1.00)	1 (−1.00)
Infidelity	2 (−0.60)	4 (−0.93)	2 (−0.50)	2 (−0.70)	1 (−1.00)	1 (−1.00)
Personal characteristics that negatively affect social harmony	2 (−0.60)	9 (−0.80)	17 (−0.05)	14 (−0.40)	9 (−0.90)	1 (−1.00)
Family separation & neglect	2 (−0.60)	17 (−0.50)	2 (−0.50)	16 (−0.20)	1 (−1.00)	25 (−0.30)
Suicidality	2 (−0.60)	17 (−0.50)	No influence	1 (−1.00)	15 (−0.50)	No influence
Lack of religious faith	2 (−0.60)	No influence	17 (−0.05)	No influence	No influence	14 (−0.50)
Misuse of technology	2 (−0.60)	No influence	14 (−0.30)	No influence	No influence	14 (−0.50)
Risk of death	2 (−0.60)	No influence	2 (−0.50)	12 (−0.50)	1 (−1.00)	No influence
Basic resource insecurity	11 (−0.50)	4 (−0.93)	No net influence	2 (−0.70)	11 (−0.80)	1 (−1.00)
Unemployment	11 (−0.50)	4 (−0.93)	No net influence	9 (−0.65)	23 (−0.30)	1 (−1.00)
Lack of affectionate, trusting, supportive family relationships	11 (−0.50)	9 (−0.80)	2 (−0.50)	9 (−0.65)	1 (−1.00)	12 (−0.93)
Low self-esteem	18 (−0.40)	1 (−1.00)	No influence	No influence	26 (−0.20)	No influence
Domestic violence	23 (−0.30)	9 (−0.80)	13 (−0.40)	9 (−0.65)	15 (−0.50)	1 (−1.00)
Poor health promotive care practices	28 (−0.05)	4 (−0.93)	15 (−0.10)	16 (−0.20)	26 (−0.20)	1 (−1.00)
Harmful gender norms	No influence	9 (−0.80)	No net influence	16 (−0.20)	No influence	1 (−1.00)
Excessive workload	18 (−0.40)	16 (−0.60)	2 (−0.50)	2 (−0.70)	22 (−0.35)	22 (−0.40)
Theft	18 (−0.40)	No influence	2 (−0.50)	16 (−0.20)	15 (−0.50)	14 (−0.50)
Bad thoughts	No influence	17 (−0.50)	2 (−0.50)	16 (−0.20)	14 (−0.70)	14 (−0.50)
Problems	11 (−0.50)	No influence	2 (−0.50)	No influence	15 (−0.50)	No influence
Unwanted pregnancies	No influence	14 (−0.70)	No influence	2 (−0.70)	11 (−0.80)	14 (−0.50)
Irresponsibility	18 (−0.40)	13 (−0.73)	No net influence	25 (−0.15)	1 (−1.00)	1 (−1.00)

*Note.* Table includes all factors that ranked among the top eight influences on men’s wellbeing in at least one of the included stakeholder maps.

**Figure 2.**
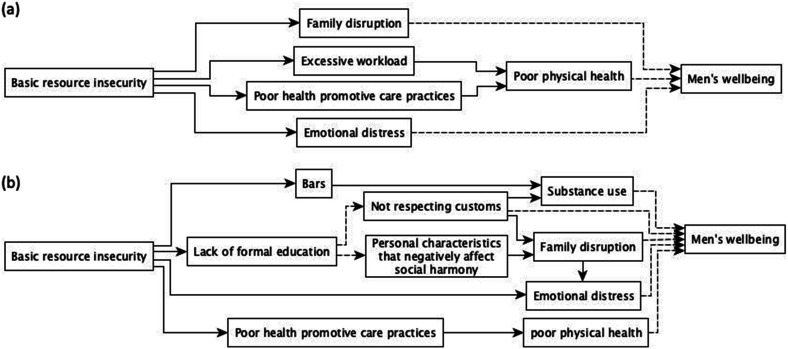
Key pathways between basic resource insecurity and men’s wellbeing, as understood by (a) adult men in Cuilco, and (b) adult men in Santiago Atitlán. *Note.* Solid lines indicate positive (promotive) relationships, and dotted lines indicate negative (inhibitory) relationships. Multiple family-level factors (infidelity; lack of affectionate, trusting, supportive family relationships; family separation & neglect; disrupted family education) were understood to contribute to pathways between basic resource insecurity and men’s wellbeing, and have been summarized as ‘family disruption.’

There were some notable differences between the regions. Men’s maps in Cuilco placed greater emphasis on low self-esteem and poor health promotive care practices, and Cuilco maps but not Santiago Atitlán maps identified harmful gender norms (machismo, unequal treatment of women) as a strong risk factor for men’s wellbeing. Santiago Atitlán maps but not Cuilco maps identified lack of religious faith, misuse of technology and “not respecting customs” as risk factors for men’s wellbeing. “Not respecting customs” included specific values and cultural practices: “losing *Xjaan”* (loss of understanding that everything is sacred, which brings negative consequences when specific customs to respect the sacred are not followed) and “harmful interpretations of rights” (referring to human rights discourse that emphasizes individual rights over collective responsibilities). In both regions, basic resource insecurity was perceived as negatively influencing men’s wellbeing (through increased emotional distress, poor health promotive care practices, poor physical health). However, in Santiago Atitlán, men’s maps portrayed basic resource insecurity as also having a positive influence on wellbeing by limiting access to formal education. Lack of access to formal education was represented as reducing wellbeing in Cuilco maps, but as having a positive influence on wellbeing in Santiago Atitlán maps. Santiago Atitlán maps suggested that local men who do not access formal education may retain more respect for customs and may be less likely to develop character traits like arrogance and disrespect, which in turn would improve wellbeing. Key pathways between basic resource insecurity and men’s wellbeing, as depicted by adult men in the two regions, are illustrated in Figure 2. A full list of factor rankings is available in Supplemental Table 2.

#### Adult Women

Adult women across both regions ranked poor physical health, emotional distress, substance use, excessive workload, and family-related factors (infidelity; lack of affectionate, trusting, and supportive family relationships) among the top risk factors for men’s wellbeing (see Table 3). Basic resource insecurity was seen as a strong risk factor for men’s wellbeing in Cuilco maps but had no net influence on men’s wellbeing in Santiago Atitlán maps. In Santiago Atitlán, the perceived negative influence of basic resource insecurity on men’s wellbeing was balanced by an equally strong perceived positive influence of this factor (through reduction of infidelity and unequal power relationships in the couple). Several factors in maps from Santiago Atitlán were perceived to have both positive and negative influences on men’s wellbeing, so little overall influence. Women’s maps in Cuilco but not Santiago Atitlán identified unwanted pregnancies and suicidality as strong risk factors for men’s wellbeing. Supplemental Table 3 gives a full list of factor rankings.

#### Terapeutas Mayas/Tradicionales

*Terapeutas* across both regions ranked substance use, poor physical health, irresponsibility, emotional distress, and factors related to family and social relations among the top influences on men’s wellbeing (see Table 3). Those in Santiago Atitlán but not Cuilco ranked social isolation, family separation and neglect, and “not respecting customs” as strong influences on men’s wellbeing. The *Terapeutas Mayas* making the maps in Santiago Atitlán specifically mentioned loss of historical memory and the discontinued use of the *tuj* (a traditional steam bath understood to have cultural, healing, and spiritual dimensions^38^) as important aspects of “not respecting customs”. *Terapeutas tradicionales* in Cuilco placed greater emphasis on harmful gender norms, domestic violence, unemployment, and poor health promotive care practices as risk factors for men’s wellbeing. See Supplemental Table 4 for a full list of factor rankings.

### Stakeholder Group Comparisons

In Santiago Atitlán, maps of men, women and *Terapeutas Mayas* all ranked poor physical health, emotional distress, substance use, and factors related to family relations among the top risk factors for men’s wellbeing (see Table 3). Suicidality, lack or religious faith, and misuse of technology were ranked more highly as risk factors in men’s maps. Maps of *Terapeutas Mayas* stressed not respecting customs, unwanted pregnancies, and irresponsibility as risk factors.

In Cuilco, all stakeholder groups ranked poor physical health, emotional distress, substance use, family-related factors, basic resource insecurity and unemployment among the most important risk factors for men’s wellbeing (see Table 3). Adult men’s maps ranked low self-esteem as a top risk for men’s wellbeing, but this factor did not feature in the maps of adult women or *Terapeutas Tradicionales*.

### Age Differences

In Santiago Atitlán, young adults and older adults created maps as well as adults. There were some age-related differences in the maps. Young adult maps emphasized social isolation, age-related family concerns (early marriage, forced marriage,) and low self-esteem as risk factors more than maps from adult and older adult groups. Young men’s maps also depicted lack of formal education as a top risk factor for wellbeing, unlike adult and older adult men’s maps, which depicted lack of formal education as having a protective influence on men’s wellbeing. Only the older men’s map ranked not respecting customs (including disconnection from identity, loss of respect for medicinal plants, loss of *tuj*, not working the land, following *ladino* advice on family planning, and “putting a price on everything”) as a top risk factor for men’s wellbeing.

## Discussion

FCM systematized local knowledge on risk and protective factors for men’s wellbeing and suggested priority concerns among remote and Indigenous communities in Guatemala. Consistent with research on Indigenous wellbeing in North America, Australia and New Zealand,^39,40^ group definitions of wellbeing reflected a holistic understanding of wellbeing across regions and stakeholder groups. Definitions emphasized social relations, hard work, capacity to meet basic needs, and physical and emotional health. Maps of all stakeholder groups suggested that top priorities for improving men’s wellbeing include promoting physical and emotional health, reducing substance use, and addressing problematic family dynamics. The consistent high ranking of these factors across maps of diverse stakeholder groups suggests that interventions targeting these issues could generate buy-in from community members of different ages and genders. Specific concerns related to family dynamics included domestic violence, infidelity, and a lack of affectionate, trusting, and supportive relationships. Comparisons between the two regions with differing degrees of Indigenous cultural continuity highlighted a greater focus on religion and loss of cultural values and practices in Santiago Atitlán. Maps from Santiago Atitlán also presented a more nuanced understanding of the influence of income and formal schooling on men’s wellbeing. Age comparisons in Santiago Atitlán highlighted greater emphasis on loss of cultural values and practices among older generations; young adults in Santiago Atitlán held views regarding formal schooling, income, and self-esteem close to adult views in Cuilco.

The importance placed on family and social relations for men’s wellbeing and emotional health raises doubts that the scale-up of individualized biomedical interventions will be sufficient to address mental health concerns in Guatemala.^3^ Research internationally suggests that intergenerational connections and trusting and supportive relationships within the family and community are essential to the wellbeing of Indigenous peoples.^5,39–41^ Indigenous scholars have argued that a focus on western mental health discourse and practices is a form of continued colonization of Indigenous peoples.^42^ This view urges the development of alternative approaches to promoting mental health that are compatible with Indigenous worldviews. There is a growing body of research on Indigenous men’s groups, primarily in Australia, which shows that spaces for men to connect with other men and engage in participatory learning and action can improve social connections, connection to cultural identity and subjective wellbeing.^43^ Future research could explore the potential of such an approach to promote the wellbeing of Indigenous and *ladino* men in Guatemala.

Comparison of maps between Santiago Atitlán and Cuilco highlighted important differences in understandings of the risk and protective factors for men’s wellbeing. A greater emphasis on harmful gender norms in Cuilco may reflect the severity of the problem or greater exposure to programming that promotes gender equality and calls attention to harmful gender norms, such as the MenCare program that has been implemented in Guatemala.^44^ Indicators of gender inequity (domestic violence, marital control) are similar across regions.^45^ While men’s groups in Santiago Atitlán did not articulate gender norms as a risk factor for wellbeing, they did highlight issues associated with harmful gender norms, including domestic violence and men’s lack of involvement in caring for their families. While most research on gender inequity and health has focused on the deleterious impacts of harmful gender norms on women, research suggests that men’s inequitable gender attitudes and behaviors also negatively impact their own mental health and wellbeing.^46^ Health interventions that encourage men to question hegemonic gender norms reduce men’s alcohol use,^47^ depression,^48^ and violence against women and girls.^49^ Nevertheless, recruitment and retention of men are persistent challenges for programs that focus on gender norms when men can’t readily identify ‘what’s in it for them’.^50^ Our findings call for practitioners and researchers to explore the feasibility and impact of interventions that engage men in critical reflection around their own wellbeing for addressing these complex social problems.

Adult men, older men and practitioners of Mayan medicine in Santiago Atitlán emphasized the importance of traditional values and cultural practices. This is consistent with research with Indigenous communities in the US, Canada, Australia, and New Zealand, highlighting the importance of cultural continuity for Indigenous mental health and wellbeing.^39,40,51,52^ Indigenous communities in Guatemala have faced multiple forces aimed at suppressing their cultural traditions and autonomy, from colonial policies to state-sponsored violence during the civil war,^53,54^ and ongoing racism and structural disadvantage.^55,56^ Researchers have documented how these forms of oppression have led Indigenous people in Guatemala to abandon their languages and traditions and disavow their Indigenous heritage.^55,57^ Rapid social and cultural change is also being accelerated by neoliberal policies that favor large international corporations at the cost of natural resources and traditional livelihoods, leading to increased reliance on wage labor and migration.^33^ Young adult groups in Santiago Atitlán did not include traditional values or cultural practices in their maps and placed greater emphasis on the importance of self-esteem for men’s wellbeing. This suggests shifting values and priorities among younger generations. A clear sense of cultural identity is associated with self-esteem and wellbeing^58^ and buffers the negative impacts of discrimination on self-esteem and mental health.^59^ One study in a town 10 miles from Santiago Atitlán found elevated rates of suicidality among participants when parents spoke an Indigenous language and they did not speak an Indigenous language themselves.^19^ Interventions that bring together youth and elders to learn from each other’s perspectives could allow youth to tap into traditional knowledge that is relevant to their own concerns. Similarly, bringing communities that have been able to maintain a greater degree of cultural continuity into dialogue with other communities in Guatemala could potentially help to build a collective understanding of the importance of culture for wellbeing.

The maps of adult men and women in Santiago Atitlán raise questions about standard development approaches that emphasize income generation and educational attainment as pathways to wellbeing in Indigenous communities. While groups in Santiago Atitlán noted the deleterious effects of resource insecurity on emotional wellbeing and physical health, they also noted how income and formal schooling could undermine social harmony, family relationships and cultural traditions. Consistent with the view of adult women’s maps, ethnographic research in Guatemala suggests that increased reliance on wage labor, coupled with exposure to hegemonic forms of masculinity through colonization, has disrupted traditional complementary roles between men and women in Indigenous Mayan communities.^60^ Indigenous people in Canada have also identified wage labor as disrupting traditional social ties and cultural practices key to wellbeing.^52^ In Ecuador, research found that income poverty was a poor indicator of subjective wellbeing for rural Indigenous communities.^61^ These findings support the call for development policies that focus on Indigenous notions of wellbeing, including a greater focus on social reciprocity, over policies that support the expansion of global capitalism.^62,63^

The views of older men’s groups in Santiago Atitlán on formal schooling echo scholars who have highlighted the role of formal schooling as a tool for cultural assimilation and subjugation of Indigenous knowledge in Guatemala.^64^ Ambivalent views of formal schooling have also been documented among Indigenous peoples in Australia, who have voiced concerns that formal schooling can be at odds with traditional forms of cultural education, despite its importance for increasing access to employment.^40^ Our findings should not be interpreted to suggest that education is unimportant for Indigenous communities. Rather, the perspectives of older men in Santiago Atitlán call for reforms to formal education systems, so that educational opportunities do not come at the cost of cultural continuity. While access to bilingual intercultural education has been official policy in Guatemala for over two decades, its implementation has been hampered by lack of consultation with local communities and persistent racist and assimilationist values within the education system.^65^

## Limitations

FCM accesses culturally salient concepts that are readily discussed in a group setting; maps are not an exhaustive summary of all participant knowledge on a topic. In this study, the maps did not include many structural influences, such as political violence or land loss, that are thought to be risk factors for men’s wellbeing. This could be because these factors are ubiquitous, and people do not identify them as causes of differences between men who are experiencing wellbeing and those who are not; because people see these structural factors as not being amenable to change; or because of social stigma attached to certain topics. Our previous research and experience in Guatemala have drawn our attention to well-founded fear around certain topics, including Indigenous forms of spirituality, that are understood to be politically polarizing. People creating the maps tended to highlight areas of personal agency and inter-personal relationships.

While we framed the mapping process around wellbeing and encouraged participants to identify promotive factors to fit with a strengths-based approach, most groups identified far more threats to men’s wellbeing. Our analysis of subsequent key informant interviews in Santiago Atitlan suggests this could partially be explained by a collective sense that community values and lifestyles are under threat in the face of rapid social, cultural and economic change. While framing the research around wellbeing alone was not sufficient to emphasize resiliency factors, we found that maintaining a focus on strengths during subsequent stages of the participatory process led these same groups to plan actions rooted in Indigenous forms of collective resiliency.^66^

We included group facilitators but not mapping participants in the thematic analysis of the concepts included in the maps due to time constraints. We did not document in-depth participant understandings of the meanings of concepts included in the maps as part of the FCM process. However, we found that shared meaning of concepts among the participants meant FCM helped groups to set priorities for collective action. We carried out subsequent key informant interviews that tapped into local histories and prototypes of wellbeing to explore in greater depth the concepts and pathways that emerged through FCM.^66^

In our experience with FCM, lack of literacy is not a bar to full participation. The facilitator writes the concepts identified by participants and reminds them of the concepts as they draw links and weight them.^30^ A challenge in this study was the broad central topic, with many causal factors identified. This led to long sessions to create the maps, and participants were tired by the time they came to weight all the links in the maps.

There are 22 unique Mayan ethnic groups in Guatemala, in addition to the *Xinca, Garífuna*, afrodescendent and *ladino* ethnic groups, with distinct languages, histories, cultural values and practices. The data we collected across two sites should not be understood to reflect the views of the Guatemalan population as a whole. Our comparison across distinct ethnic contexts, ages and genders, as well as the incorporation of the perspectives of *Terapeutas Mayas/Tradicionales*, sheds light on issues likely to be salient across many ethnic contexts and stakeholder groups.

## Conclusions

Creating meaningful and effective strategies to promote health and wellbeing in Indigenous contexts requires understanding priorities from the worldview of Indigenous communities themselves. Fuzzy cognitive mapping offered important insights into factors that are relevant to men’s wellbeing in Guatemala from the perspectives of different stakeholders in remote and Indigenous communities. Maps highlighted the importance of maintaining cultural traditions and promoting positive family and community relations for Indigenous men’s wellbeing. Maps also identified the mixed influence of factors such as income and formal schooling on men’s wellbeing. The findings call for alternative development models that strengthen rather than detract from Indigenous knowledge systems and ways of life.

## Supplemental Material

Supplemental Material - Community Views of Determinants of Men’s Wellbeing in Guatemala: A Study Using Fuzzy Cognitive Mapping Supplemental Material for Community Views of Determinants of Men’s Wellbeing in Guatemala: A Study Using Fuzzy Cognitive Mapping by Katherine W. Pizarro, Anne M. Chomat, Diego P. Quieju, Bernardo Y. López, Iván Sarmiento, Nicholas LeBel, Chloe Mancini, Neil Andersson, Danielle Groleau, and Anne Cockcroft in Community Health Equity Research and Policy

Supplemental Material - Community Views of Determinants of Men’s Wellbeing in Guatemala: A Study Using Fuzzy Cognitive Mapping Supplemental Material for Community Views of Determinants of Men’s Wellbeing in Guatemala: A Study Using Fuzzy Cognitive Mapping by Katherine W. Pizarro, Anne M. Chomat, Diego P. Quieju, Bernardo Y. López, Iván Sarmiento, Nicholas LeBel, Chloe Mancini, Neil Andersson, Danielle Groleau, and Anne Cockcroft in Community Health Equity Research and Policy

Supplemental Material - Community Views of Determinants of Men’s Wellbeing in Guatemala: A Study Using Fuzzy Cognitive Mapping Supplemental Material for Community Views of Determinants of Men’s Wellbeing in Guatemala: A Study Using Fuzzy Cognitive Mapping by Katherine W. Pizarro, Anne M. Chomat, Diego P. Quieju, Bernardo Y. López, Iván Sarmiento, Nicholas LeBel, Chloe Mancini, Neil Andersson, Danielle Groleau, and Anne Cockcroft in Community Health Equity Research and Policy

Supplemental Material - Community Views of Determinants of Men’s Wellbeing in Guatemala: A Study Using Fuzzy Cognitive Mapping Supplemental Material for Community Views of Determinants of Men’s Wellbeing in Guatemala: A Study Using Fuzzy Cognitive Mapping by Katherine W. Pizarro, Anne M. Chomat, Diego P. Quieju, Bernardo Y. López, Iván Sarmiento, Nicholas LeBel, Chloe Mancini, Neil Andersson, Danielle Groleau, and Anne Cockcroft in Community Health Equity Research and Policy

Supplemental Material - Community Views of Determinants of Men’s Wellbeing in Guatemala: A Study Using Fuzzy Cognitive Mapping Supplemental Material for Community Views of Determinants of Men’s Wellbeing in Guatemala: A Study Using Fuzzy Cognitive Mapping by Katherine W. Pizarro, Anne M. Chomat, Diego P. Quieju, Bernardo Y. López, Iván Sarmiento, Nicholas LeBel, Chloe Mancini, Neil Andersson, Danielle Groleau, and Anne Cockcroft in Community Health Equity Research and Policy
