# Supplementary material for: Community Views of Determinants of Men’s Wellbeing in Guatemala: A Study Using Fuzzy Cognitive Mapping
Source: Community Health Equity Res Policy. 2025 Jan 15;46(2):157–69. doi: 10.1177/2752535X241312378 (PMC12627251; doi:10.1177/2752535X241312378)
Supplement: Supplemental Material - Community Views of Determinants of Men’s Wellbeing in Guatemala: A Study Using Fuzzy Cognitive Mapping [file sj-pdf-1-qch-10.1177_2752535X241312378.pdf]

Supplementary table 1. Factors included in FCM analysis and description of each factor

Factor name	Description
Bad thoughts	Includes bad thoughts, negative desires, negative attitude
Bars	Only includes bars
Basic resource insecurity	Includes poverty, economic problems, insufficient basic resources (i.e. clothing, housing, food), not having money
Child labor	Only includes child labor
Early dating/marriage/ sex/pregnancy	Includes dating, sex, pregnancy and marriage at a young age
Disrupted family education	Includes lack of family education, loss of ancestral knowledge, lack of parental discipline, child disobedience
Domestic violence	Includes physical and sexual violence
Emotional distress	Includes descriptions of distress (i.e. worries, anger), specific local idioms of distress, unhappiness, mental illness categories
Excessive workload	Includes excess work, lack of time due to work, heavy lifting
Family separation & neglect	Includes family separation, divorce, lack of family unity, neglectful parenting, abandonment by spouse
Forced marriage	Only includes forced marriage
Harmful gender norms	Includes machismo, gender norms, unequal treatment of women
Infertility	Includes infertility, not having descendants
Infidelity	Includes men's and women's infidelity
Irresponsibility	Includes laziness, irresponsibility, vagrancy, bad decision making, lack of plans, lack of purpose, poor economic administration
Lack of access to health services and health information	Includes difficulty accessing medication and health information
Lack of affectionate, trusting, supportive family relationships	Includes family problems, verbal violence, distrust, jealousy, lack of love, lack of understanding, poor communication, lack of partner support, lack of attention within the family
Lack of formal education	Includes not having formal education/schooling
Lack of religious faith	Includes not having faith in God, lack of spiritual orientation
Low self-esteem	Includes low self-esteem, lack of self-confidence, feelings of inferiority
Migration	Only includes migration
Misuse of technology	Includes violent and pornographic media content, overuse of cell phones, use of social media for infidelity
Negative social influences	Includes bad examples, bad friends
Not communicating feelings/ seeking support	Includes not talking about problems, not seeking help
Not respecting customs	Includes loss of traditional forms of health promotion and healing (e.g. *tuj* (steam bath), herbal remedies), disconnection from identity,

	loss of historical memory, loss of traditional dress, loss of traditional values (e.g. following advice from ladinos about family planning, "losing *Xjaan"* (not respecting the sacred), "harmful interpretations of rights," "putting a price on everything"), changing ways of life (e.g. not working the land)
Not sleeping well	Includes insomnia, not sleeping well
Personal characteristics that negatively affect social harmony	Includes bad character, selfishness, disrespect, dishonesty, pride, lies, disobedience, lack of patience, lack of empathy
Poor health promotive care practices	Includes poor diet, poor hygiene, misuse of insecticides
Poor physical health	Includes illness, physical pain/weakness, injury, disability, malnutrition, gastritis, sexually transmitted infections, HIV
Prison	Includes prison
Problems	Only includes problems
Risk of death	Only includes risk of death
Self-care	Includes improving one's way of life, personal grooming
Social isolation	Includes social rejection, discrimination, isolation, lack of group participation, loneliness
Sports/recreation	Includes sports, recreation, recreational spaces
Substance use	Includes alcohol use, drug use, addiction, "vices"
Suicidality	Only includes suicidality
Taking care of the environment	Only includes taking care of the environment
Theft	Only includes theft
Unemployment	Includes not having work, limited employment opportunities
Unequal power relationship in couple	Includes men being possessive and exerting economic control
Unwanted pregnancies	Includes unwanted pregnancies, lack of family planning, having too many children
Witchcraft	Only includes witchcraft

Note: Some factors included a minority of concepts with opposing meanings (e.g. absence of illness within poor physical health). In these instances, maps were corrected to preserve the intended relationships between factors when replacing concepts with factor names.
