# Supplementary material for: Community Views of Determinants of Men’s Wellbeing in Guatemala: A Study Using Fuzzy Cognitive Mapping
Source: Community Health Equity Res Policy. 2025 Jan 15;46(2):157–69. doi: 10.1177/2752535X241312378 (PMC12627251; doi:10.1177/2752535X241312378)
Supplement: Supplemental Material - Community Views of Determinants of Men’s Wellbeing in Guatemala: A Study Using Fuzzy Cognitive Mapping [file sj-pdf-2-qch-10.1177_2752535X241312378.pdf]

Supplementary table 2. Ranking of factors according to net causal influence on men's wellbeing in maps from adult men in the two regions

	Santiago Atitlán		Cuilco	
**Factor**	**Rank (Net influence)**	**# of maps that included the factor (of 2)**	**Rank (Net influence)**	**# of maps that included the factor (of 2)**
Poor physical health	1 (-0.95)	2	4 (-0.93)	2
Emotional distress	2 (-0.60)	2	1 (-1.00)	2
Substance use	2 (-0.60)	2	1 (-1.00)	2
Infidelity	2 (-0.60)	2	4 (-0.93)	2
Personal characteristics that negatively affect social harmony	2 (-0.60)	2	9 (-0.80)	2
Family separation & neglect	2 (-0.60)	2	17 (-0.50)	1
Suicidality	2 (-0.60)	2	17 (-0.50)	1
Lack of religious faith	2 (-0.60)	2	No influence	0
Misuse of technology	2 (-0.60)	2	No influence	0
Risk of death	2 (-0.60)	2	No influence	0
Basic resource insecurity	11 (-0.50)	2	4 (-0.93)	2
Unemployment	11 (-0.50)	2	4 (-0.93)	2
Lack of affectionate, trusting, supportive family relationships	11 (-0.50)	2	9 (-0.80)	2
Problems	11 (-0.50)	1	No influence	0
Bars	11 (-0.50)	1	No influence	0
Lack of access to health services and health information	16 (-0.45)	1	No influence	0
Social isolation	16 (-0.45)	2	No influence	0
Low self-esteem	18 (-0.40)	2	1 (-1.00)	2
Irresponsibility	18 (-0.40)	2	13 (-0.73)	2
Excessive workload	18 (-0.40)	2	16 (-0.60)	2
Negative social influences	18 (-0.40)	2	23 (-0.30)	1
Theft	18 (-0.40)	2	No influence	0
Domestic violence	23 (-0.30)	2	9 (-0.80)	2
Lack of formal education	24 (0.10)	1	17 (-0.50)	1
Dating/marriage/sex/ pregnancy at a young age	24 (-0.10)	1	No influence	0

Disrupted family education	24 (-0.10)	1	No influence	0
Not respecting customs	24 (-0.10)	1	No influence	0
Poor health promotive care practices	28 (-0.05)	1	4 (-0.93)	2
Harmful gender norms	No influence	0	9 (-0.80)	2
Not sleeping well	No influence	0	14 (-0.70)	2
Unwanted pregnancies	No influence	0	14 (-0.70)	2
Not communicating feelings/ seeking support	No influence	0	17 (-0.50)	1
Migration	No influence	0	17 (-0.50)	1
Bad thoughts	No influence	0	17 (-0.50)	1
Taking care of the environment	No influence	0	23 (0.30)	1
Sports/recreation	No influence	0	23 (0.30)	1
