# Supplementary material for: Community Views of Determinants of Men’s Wellbeing in Guatemala: A Study Using Fuzzy Cognitive Mapping
Source: Community Health Equity Res Policy. 2025 Jan 15;46(2):157–69. doi: 10.1177/2752535X241312378 (PMC12627251; doi:10.1177/2752535X241312378)
Supplement: Supplemental Material - Community Views of Determinants of Men’s Wellbeing in Guatemala: A Study Using Fuzzy Cognitive Mapping [file sj-pdf-3-qch-10.1177_2752535X241312378.pdf]

Supplementary table 3. Ranking of factors according to net causal influence on men's wellbeing in maps from adult women in the two regions

	**Santiago Atitlán**		**Cuilco**	
**Factor**	**Rank (Net influence)**	**# of maps that included the factor (of 2)**	**Rank (Net influence)**	**# of maps that included the factor (of 2)**
Poor physical health	1 (-0.60)	2	2 (-0.70)	2
Emotional distress	2 (-0.50)	2	2 (-0.70)	2
Excessive workload	2 (-0.50)	1	2 (-0.70)	2
Substance use	2 (-0.50)	2	2 (-0.70)	2
Infidelity	2 (-0.50)	2	2 (-0.70)	2
Lack of affectionate, trusting, supportive family relationships	2 (-0.50)	2	9 (-0.65)	2
Risk of death	2 (-0.50)	2	12 (-0.50)	1
Family separation & neglect	2 (-0.50)	2	16 (-0.20)	1
Theft	2 (-0.50)	2	16 (-0.20)	1
Bad thoughts	2 (-0.50)	2	16 (-0.20)	1
Problems	2 (-0.50)	2	No influence	0
Disrupted family education	12 (-0.45)	1	No influence	0
Domestic violence	13 (-0.40)	2	9 (-0.65)	2
Misuse of technology	14 (-0.30)	1	No influence	0
Poor health promotive care practices	15 (-0.10)	1	16 (-0.20)	1
Social isolation	15 (-0.10)	1	No influence	0
Personal characteristics that negatively affect social harmony	17 (-0.05)	1	14 (-0.40)	1
Lack of religious faith	17 (-0.05)	1	No influence	0
Suicidality	No influence	0	1 (-1.00)	2
Basic resource insecurity	No net influence	2	2 (-0.70)	2
Unwanted pregnancies	No influence	0	2 (-0.70)	2
Unemployment	No net influence	1	9 (-0.65)	2
Lack of formal education	No net influence	1	13 (-0.45)	1
Negative social influences	No net influence	2	15 (-0.35)	2

Migration	No net influence	1	16 (-0.20)	1
Harmful gender norms	No net influence	1	16 (-0.20)	1
Prison	No influence	0	16 (-0.20)	1
Self-care	No influence	0	16 (0.20)	1
Sports/recreation	No influence	0	16 (0.20)	1
Irresponsibility	No net influence	2	25 (-0.15)	1
Child labor	No net influence	1	No influence	0
Not communicating feelings/ seeking support	No net influence	1	No influence	0
Unequal power relationship in couple	No net influence	1	No influence	0
