# Supplementary material for: Community Views of Determinants of Men’s Wellbeing in Guatemala: A Study Using Fuzzy Cognitive Mapping
Source: Community Health Equity Res Policy. 2025 Jan 15;46(2):157–69. doi: 10.1177/2752535X241312378 (PMC12627251; doi:10.1177/2752535X241312378)
Supplement: Supplemental Material - Community Views of Determinants of Men’s Wellbeing in Guatemala: A Study Using Fuzzy Cognitive Mapping [file sj-pdf-4-qch-10.1177_2752535X241312378.pdf]

Supplementary table 4. Ranking of factors according to net causal influence on men's wellbeing in maps from *Terapeutas Mayas/tradicionales* in the two regions

	**Santiago Atitlán**		**Cuilco**	
**Factor**	**Rank (Net influence)**	**# of maps that included the factor (of 2)**	**Rank (Net influence)**	**# of maps that included the factor (of 2)**
Substance use	1 (-1.00)	2	1 (-1.00)	2
Poor physical health	1 (-1.00)	2	1 (-1.00)	2
Infidelity	1 (-1.00)	2	1 (-1.00)	2
Irresponsibility	1 (-1.00)	2	1 (-1.00)	2
Emotional distress	1 (-1.00)	2	11 (-0.98)	2
Lack of affectionate, trusting, supportive family relationships	1 (-1.00)	2	12 (-0.93)	2
Family separation & neglect	1 (-1.00)	2	25 (-0.30)	1
Risk of death	1 (-1.00)	2	No influence	0
Personal characteristics that negatively affect social harmony	9 (-0.90)	2	1 (-1.00)	2
Social isolation	9 (-0.90)	2	0	0
Basic resource insecurity	11 (-0.80)	2	1 (-1.00)	2
Unwanted pregnancies	11 (-0.80)	2	14 (-0.50)	1
Not respecting customs	11 (-0.80)	2	No influence	0
Bad thoughts	14 (-0.70)	2	14 (-0.50)	1
Domestic violence	15 (-0.50)	1	1 (-1.00)	2
Negative social influences	15 (-0.50)	2	12 (-0.93)	2
Theft	15 (-0.50)	1	14 (-0.50)	1
Unequal power relationship in couple	15 (-0.50)	1	No influence	0
Not sleeping well	15 (-0.50)	1	No influence	0
Problems	15 (-0.50)	1	No influence	0
Suicidality	15 (-0.50)	1	No influence	0
Excessive workload	22 (-0.35)	1	22 (-0.40)	1
Unemployment	23 (-0.30)	1	1 (-1.00)	2
Lack of access to health services and health information	23 (-0.30)	1	No influence	0
Infertility	23 (-0.30)	1	No influence	0

Poor health promotive care practices	26 (-0.20)	1	1 (-1.00)	2
Lack of formal education	26 (-0.20)	1	25 (-0.30)	1
Low self-esteem	26 (-0.20)	1	No influence	0
Harmful gender norms	No influence	0	1 (-1.00)	2
Self-care	No influence	0	14 (0.50)	1
Taking care of the environment	No influence	0	14 (0.50)	1
Lack of religious faith	No influence	0	14 (-0.50)	1
Misuse of technology	No influence	0	14 (-0.50)	1
Prison	No influence	0	21 (-0.43)	1
Migration	No influence	0	22 (-0.40)	1
Sports/recreation	No influence	0	22 (0.40)	1
