# Supplementary material for: Community Views of Determinants of Men’s Wellbeing in Guatemala: A Study Using Fuzzy Cognitive Mapping
Source: Community Health Equity Res Policy. 2025 Jan 15;46(2):157–69. doi: 10.1177/2752535X241312378 (PMC12627251; doi:10.1177/2752535X241312378)
Supplement: Supplemental Material - Community Views of Determinants of Men’s Wellbeing in Guatemala: A Study Using Fuzzy Cognitive Mapping [file sj-pdf-5-qch-10.1177_2752535X241312378.pdf]

Supplementary table 5. List of all factors, connections, and weights in individual maps by location and stakeholder group.

Community	Map	Source Node	Target Node	Weight
Santiago Atitlán, community 1	Adult men	Emotional distress	Men's wellbeing	-4.8
Santiago Atitlán, community 1	Adult men	Emotional distress	Social isolation	4.5
Santiago Atitlán, community 1	Adult men	Emotional distress	Family separation & neglect	4
Santiago Atitlán, community 1	Adult men	Emotional distress	Domestic violence	5
Santiago Atitlán, community 1	Adult men	Emotional distress	Lack of affectionate, trusting, supportive family relationships	5
Santiago Atitlán, community 1	Adult men	Emotional distress	Irresponsibility	5
Santiago Atitlán, community 1	Adult men	Emotional distress	Personal characteristics that negatively affect social harmony	4.67
Santiago Atitlán, community 1	Adult men	Emotional distress	Risk of death	5
Santiago Atitlán, community 1	Adult men	Emotional distress	Substance use	5
Santiago Atitlán, community 1	Adult men	Emotional distress	Suicidality	3
Santiago Atitlán, community 1	Adult men	Lack of access to health services and health information	Men's wellbeing	-3
Santiago Atitlán, community 1	Adult men	Lack of access to health services and health information	Poor physical health	5
Santiago Atitlán, community 1	Adult men	Social isolation	Emotional distress	4.5
Santiago Atitlán, community 1	Adult men	Social isolation	Suicidality	4
Santiago	Adult men	Negative social	Personal characteristics	4

Atitlán, community 1		influences	that negatively affect social harmony	
Santiago Atitlán, community 1	Adult men	Negative social influences	Disrupted family education	5
Santiago Atitlán, community 1	Adult men	Negative social influences	Infidelity	5
Santiago Atitlán, community 1	Adult men	Early dating/marriage/sex/pregnancy	Low self-esteem	5
Santiago Atitlán, community 1	Adult men	Early dating/marriage/sex/pregnancy	Infidelity	5
Santiago Atitlán, community 1	Adult men	Basic resource insecurity	Emotional distress	5
Santiago Atitlán, community 1	Adult men	Basic resource insecurity	Lack of access to health services and health information	4
Santiago Atitlán, community 1	Adult men	Basic resource insecurity	Unemployment	5
Santiago Atitlán, community 1	Adult men	Basic resource insecurity	Theft	5
Santiago Atitlán, community 1	Adult men	Basic resource insecurity	Poor health promotive care practices	5
Santiago Atitlán, community 1	Adult men	Basic resource insecurity	Lack of formal education	5
Santiago Atitlán, community 1	Adult men	Unemployment	Basic resource insecurity	5
Santiago Atitlán, community 1	Adult men	Unemployment	Theft	5
Santiago Atitlán, community 1	Adult men	Family separation & neglect	Emotional distress	5
Santiago Atitlán, community 1	Adult men	Domestic violence	Men's wellbeing	-4
Santiago Atitlán,	Adult men	Domestic violence	Social isolation	4

community 1				
Santiago Atitlán, community 1	Adult men	Domestic violence	Family separation & neglect	3
Santiago Atitlán, community 1	Adult men	Lack of affectionate, trusting, supportive family relationships	Emotional distress	4
Santiago Atitlán, community 1	Adult men	Irresponsibility	Men's wellbeing	-4.5
Santiago Atitlán, community 1	Adult men	Irresponsibility	Emotional distress	5
Santiago Atitlán, community 1	Adult men	Irresponsibility	Negative social influences	5
Santiago Atitlán, community 1	Adult men	Irresponsibility	Early dating/marriage/sex/pregnancy	5
Santiago Atitlán, community 1	Adult men	Irresponsibility	Personal characteristics that negatively affect social harmony	5
Santiago Atitlán, community 1	Adult men	Irresponsibility	Disrupted family education	5
Santiago Atitlán, community 1	Adult men	Irresponsibility	Misuse of technology	5
Santiago Atitlán, community 1	Adult men	Irresponsibility	Poor physical health	-4
Santiago Atitlán, community 1	Adult men	Theft	Domestic violence	5
Santiago Atitlán, community 1	Adult men	Theft	Risk of death	5
Santiago Atitlán, community 1	Adult men	Personal characteristics that negatively affect social harmony	Emotional distress	5
Santiago Atitlán, community 1	Adult men	Personal characteristics that negatively affect social harmony	Social isolation	4
Santiago Atitlán, community 1	Adult men	Personal characteristics that negatively affect social harmony	Family separation & neglect	5

Santiago Atitlán, community 1	Adult men	Personal characteristics that negatively affect social harmony	Irresponsibility	4.5
Santiago Atitlán, community 1	Adult men	Personal characteristics that negatively affect social harmony	Not respecting customs	5
Santiago Atitlán, community 1	Adult men	Personal characteristics that negatively affect social harmony	Misuse of technology	5
Santiago Atitlán, community 1	Adult men	Personal characteristics that negatively affect social harmony	Poor physical health	3
Santiago Atitlán, community 1	Adult men	Poor health promotive care practices	Emotional distress	4
Santiago Atitlán, community 1	Adult men	Poor health promotive care practices	Poor physical health	5
Santiago Atitlán, community 1	Adult men	Lack of formal education	Personal characteristics that negatively affect social harmony	-5
Santiago Atitlán, community 1	Adult men	Lack of formal education	Disrupted family education	-4.5
Santiago Atitlán, community 1	Adult men	Lack of formal education	Not respecting customs	-5
Santiago Atitlán, community 1	Adult men	Lack of religious faith	Emotional distress	5
Santiago Atitlán, community 1	Adult men	Lack of religious faith	Basic resource insecurity	3
Santiago Atitlán, community 1	Adult men	Lack of religious faith	Personal characteristics that negatively affect social harmony	5
Santiago Atitlán, community 1	Adult men	Lack of religious faith	Not respecting customs	5
Santiago Atitlán, community 1	Adult men	Disrupted family education	Lack of access to health services and health information	4
Santiago Atitlán, community 1	Adult men	Disrupted family education	Early dating/marriage/sex/pregnancy	5
Santiago	Adult men	Disrupted family	Irresponsibility	4.67

Atitlán, community 1		education		
Santiago Atitlán, community 1	Adult men	Disrupted family education	Personal characteristics that negatively affect social harmony	4.67
Santiago Atitlán, community 1	Adult men	Disrupted family education	Lack of religious faith	5
Santiago Atitlán, community 1	Adult men	Disrupted family education	Low self-esteem	5
Santiago Atitlán, community 1	Adult men	Disrupted family education	Poor physical health	4
Santiago Atitlán, community 1	Adult men	Not respecting customs	Men's wellbeing	-5
Santiago Atitlán, community 1	Adult men	Not respecting customs	Emotional distress	5
Santiago Atitlán, community 1	Adult men	Not respecting customs	Negative social influences	5
Santiago Atitlán, community 1	Adult men	Not respecting customs	Early dating/marriage/sex/pregnancy	5
Santiago Atitlán, community 1	Adult men	Not respecting customs	Personal characteristics that negatively affect social harmony	5
Santiago Atitlán, community 1	Adult men	Not respecting customs	Disrupted family education	5
Santiago Atitlán, community 1	Adult men	Not respecting customs	Substance use	5
Santiago Atitlán, community 1	Adult men	Not respecting customs	Infidelity	5
Santiago Atitlán, community 1	Adult men	Low self-esteem	Lack of affectionate, trusting, supportive family relationships	5
Santiago Atitlán, community 1	Adult men	Low self-esteem	Infidelity	4
Santiago Atitlán,	Adult men	Misuse of technology	Infidelity	5

community 1				
Santiago Atitlán, community 1	Adult men	Poor physical health	Men's wellbeing	-4.5
Santiago Atitlán, community 1	Adult men	Poor physical health	Risk of death	3
Santiago Atitlán, community 1	Adult men	Risk of death	Men's wellbeing	-5
Santiago Atitlán, community 1	Adult men	Substance use	Men's wellbeing	-5
Santiago Atitlán, community 1	Adult men	Substance use	Family separation & neglect	5
Santiago Atitlán, community 1	Adult men	Substance use	Domestic violence	5
Santiago Atitlán, community 1	Adult men	Substance use	Theft	4
Santiago Atitlán, community 1	Adult men	Substance use	Suicidality	4
Santiago Atitlán, community 1	Adult men	Suicidality	Men's wellbeing	-5
Santiago Atitlán, community 1	Adult men	Infidelity	Family separation & neglect	5
Santiago Atitlán, community 1	Adult women	Emotional distress	Men's wellbeing	-5
Santiago Atitlán, community 1	Adult women	Emotional distress	Poor physical health	4
Santiago Atitlán, community 1	Adult women	Emotional distress	Risk of death	5
Santiago Atitlán, community 1	Adult women	Social isolation	Men's wellbeing	-5
Santiago Atitlán, community 1	Adult women	Social isolation	Emotional distress	5

Santiago Atitlán, community 1	Adult women	Social isolation	Negative social influences	5
Santiago Atitlán, community 1	Adult women	Social isolation	Infidelity	5
Santiago Atitlán, community 1	Adult women	Negative social influences	Family separation & neglect	4
Santiago Atitlán, community 1	Adult women	Negative social influences	Irresponsibility	4
Santiago Atitlán, community 1	Adult women	Basic resource insecurity	Excessive workload	5
Santiago Atitlán, community 1	Adult women	Basic resource insecurity	Lack of formal education	5
Santiago Atitlán, community 1	Adult women	Basic resource insecurity	Poor physical health	5
Santiago Atitlán, community 1	Adult women	Basic resource insecurity	Infidelity	-5
Santiago Atitlán, community 1	Adult women	Family separation & neglect	Emotional distress	5
Santiago Atitlán, community 1	Adult women	Family separation & neglect	Lack of affectionate, trusting, supportive family relationships	4
Santiago Atitlán, community 1	Adult women	Family separation & neglect	Problems	5
Santiago Atitlán, community 1	Adult women	Family separation & neglect	Substance use	5
Santiago Atitlán, community 1	Adult women	Domestic violence	Poor physical health	4
Santiago Atitlán, community 1	Adult women	Domestic violence	Risk of death	3
Santiago Atitlán, community 1	Adult women	Lack of affectionate, trusting, supportive family relationships	Emotional distress	5
Santiago	Adult women	Lack of affectionate,	Family separation &	5

Atitlán, community 1		trusting, supportive family relationships	neglect	
Santiago Atitlán, community 1	Adult women	Lack of affectionate, trusting, supportive family relationships	Disrupted family education	3
Santiago Atitlán, community 1	Adult women	Irresponsibility	Basic resource insecurity	5
Santiago Atitlán, community 1	Adult women	Theft	Risk of death	5
Santiago Atitlán, community 1	Adult women	Bad thoughts	Emotional distress	5
Santiago Atitlán, community 1	Adult women	Bad thoughts	Misuse of technology	4
Santiago Atitlán, community 1	Adult women	Personal characteristics that negatively affect social harmony	Emotional distress	4.5
Santiago Atitlán, community 1	Adult women	Personal characteristics that negatively affect social harmony	Social isolation	5
Santiago Atitlán, community 1	Adult women	Personal characteristics that negatively affect social harmony	Family separation & neglect	4
Santiago Atitlán, community 1	Adult women	Personal characteristics that negatively affect social harmony	Lack of affectionate, trusting, supportive family relationships	4
Santiago Atitlán, community 1	Adult women	Personal characteristics that negatively affect social harmony	Irresponsibility	4.5
Santiago Atitlán, community 1	Adult women	Personal characteristics that negatively affect social harmony	Theft	4
Santiago Atitlán, community 1	Adult women	Personal characteristics that negatively affect social harmony	Disrupted family education	5
Santiago Atitlán, community 1	Adult women	Personal characteristics that negatively affect social harmony	Problems	5
Santiago Atitlán, community 1	Adult women	Personal characteristics that negatively affect social harmony	Substance use	5
Santiago Atitlán,	Adult women	Personal characteristics that negatively affect	Infidelity	4

community 1		social harmony		
Santiago Atitlán, community 1	Adult women	Excessive workload	Family separation & neglect	5
Santiago Atitlán, community 1	Adult women	Excessive workload	Poor physical health	4
Santiago Atitlán, community 1	Adult women	Lack of formal education	Irresponsibility	4
Santiago Atitlán, community 1	Adult women	Lack of religious faith	Men's wellbeing	-5
Santiago Atitlán, community 1	Adult women	Lack of religious faith	Negative social influences	5
Santiago Atitlán, community 1	Adult women	Lack of religious faith	Family separation & neglect	5
Santiago Atitlán, community 1	Adult women	Lack of religious faith	Lack of affectionate, trusting, supportive family relationships	5
Santiago Atitlán, community 1	Adult women	Lack of religious faith	Personal characteristics that negatively affect social harmony	5
Santiago Atitlán, community 1	Adult women	Disrupted family education	Emotional distress	4.5
Santiago Atitlán, community 1	Adult women	Disrupted family education	Excessive workload	3
Santiago Atitlán, community 1	Adult women	Misuse of technology	Infidelity	3
Santiago Atitlán, community 1	Adult women	Poor physical health	Men's wellbeing	-3
Santiago Atitlán, community 1	Adult women	Poor physical health	Risk of death	5
Santiago Atitlán, community 1	Adult women	Risk of death	Men's wellbeing	-5
Santiago Atitlán, community 1	Adult women	Problems	Men's wellbeing	-5

Santiago Atitlán, community 1	Adult women	Problems	Emotional distress	4
Santiago Atitlán, community 1	Adult women	Substance use	Men's wellbeing	-5
Santiago Atitlán, community 1	Adult women	Substance use	Emotional distress	4.71
Santiago Atitlán, community 1	Adult women	Substance use	Domestic violence	5
Santiago Atitlán, community 1	Adult women	Substance use	Lack of affectionate, trusting, supportive family relationships	4
Santiago Atitlán, community 1	Adult women	Substance use	Theft	5
Santiago Atitlán, community 1	Adult women	Substance use	Poor physical health	5
Santiago Atitlán, community 1	Adult women	Substance use	Risk of death	5
Santiago Atitlán, community 1	Adult women	Infidelity	Problems	5
Santiago Atitlán, community 1	Adult women	Infidelity	Substance use	5
Santiago Atitlán, community 1	*Terapeutas Mayas*	Emotional distress	Men's wellbeing	-3.33
Santiago Atitlán, community 1	*Terapeutas Mayas*	Emotional distress	Social isolation	1
Santiago Atitlán, community 1	*Terapeutas Mayas*	Emotional distress	Personal characteristics that negatively affect social harmony	3
Santiago Atitlán, community 1	*Terapeutas Mayas*	Emotional distress	Poor physical health	4
Santiago Atitlán, community 1	*Terapeutas Mayas*	Emotional distress	Risk of death	5
Santiago	*Terapeutas*	Emotional distress	Substance use	4

Atitlán, community 1	*Mayas*			
Santiago Atitlán, community 1	*Terapeutas Mayas*	Emotional distress	Suicidality	5
Santiago Atitlán, community 1	*Terapeutas Mayas*	Lack of access to health services and health information	Unwanted pregnancies	3
Santiago Atitlán, community 1	*Terapeutas Mayas*	Social isolation	Emotional distress	4
Santiago Atitlán, community 1	*Terapeutas Mayas*	Social isolation	Personal characteristics that negatively affect social harmony	3
Santiago Atitlán, community 1	*Terapeutas Mayas*	Basic resource insecurity	Men's wellbeing	-1
Santiago Atitlán, community 1	*Terapeutas Mayas*	Basic resource insecurity	Emotional distress	3
Santiago Atitlán, community 1	*Terapeutas Mayas*	Basic resource insecurity	Unemployment	5
Santiago Atitlán, community 1	*Terapeutas Mayas*	Basic resource insecurity	Lack of formal education	4
Santiago Atitlán, community 1	*Terapeutas Mayas*	Basic resource insecurity	Not respecting customs	5
Santiago Atitlán, community 1	*Terapeutas Mayas*	Basic resource insecurity	Low self-esteem	3
Santiago Atitlán, community 1	*Terapeutas Mayas*	Basic resource insecurity	Poor physical health	3
Santiago Atitlán, community 1	*Terapeutas Mayas*	Unemployment	Basic resource insecurity	5
Santiago Atitlán, community 1	*Terapeutas Mayas*	Family separation & neglect	Problems	5
Santiago Atitlán, community 1	*Terapeutas Mayas*	Lack of affectionate, trusting, supportive family relationships	Emotional distress	5
Santiago Atitlán,	*Terapeutas Mayas*	Lack of affectionate, trusting, supportive	Social isolation	5

community 1		family relationships		
Santiago Atitlán, community 1	*Terapeutas Mayas*	Lack of affectionate, trusting, supportive family relationships	Personal characteristics that negatively affect social harmony	3
Santiago Atitlán, community 1	*Terapeutas Mayas*	Irresponsibility	Basic resource insecurity	3
Santiago Atitlán, community 1	*Terapeutas Mayas*	Irresponsibility	Risk of death	5
Santiago Atitlán, community 1	*Terapeutas Mayas*	Bad thoughts	Low self-esteem	3
Santiago Atitlán, community 1	*Terapeutas Mayas*	Personal characteristics that negatively affect social harmony	Men's wellbeing	-4
Santiago Atitlán, community 1	*Terapeutas Mayas*	Personal characteristics that negatively affect social harmony	Emotional distress	2
Santiago Atitlán, community 1	*Terapeutas Mayas*	Personal characteristics that negatively affect social harmony	Social isolation	1
Santiago Atitlán, community 1	*Terapeutas Mayas*	Personal characteristics that negatively affect social harmony	Negative social influences	3
Santiago Atitlán, community 1	*Terapeutas Mayas*	Personal characteristics that negatively affect social harmony	Irresponsibility	2.5
Santiago Atitlán, community 1	*Terapeutas Mayas*	Personal characteristics that negatively affect social harmony	Bad thoughts	3
Santiago Atitlán, community 1	*Terapeutas Mayas*	Poor health promotive care practices	Poor physical health	2
Santiago Atitlán, community 1	*Terapeutas Mayas*	Unwanted pregnancies	Emotional distress	3
Santiago Atitlán, community 1	*Terapeutas Mayas*	Lack of formal education	Low self-esteem	2
Santiago Atitlán, community 1	*Terapeutas Mayas*	Not respecting customs	Poor physical health	3
Santiago Atitlán, community 1	*Terapeutas Mayas*	Low self-esteem	Emotional distress	2

Santiago Atitlán, community 1	*Terapeutas Mayas*	Low self-esteem	Irresponsibility	2
Santiago Atitlán, community 1	*Terapeutas Mayas*	Low self-esteem	Poor health promotive care practices	2
Santiago Atitlán, community 1	*Terapeutas Mayas*	Poor physical health	Men's wellbeing	-5
Santiago Atitlán, community 1	*Terapeutas Mayas*	Poor physical health	Emotional distress	5
Santiago Atitlán, community 1	*Terapeutas Mayas*	Poor physical health	Risk of death	5
Santiago Atitlán, community 1	*Terapeutas Mayas*	Infertility	Men's wellbeing	-2
Santiago Atitlán, community 1	*Terapeutas Mayas*	Infertility	Emotional distress	3
Santiago Atitlán, community 1	*Terapeutas Mayas*	Risk of death	Men's wellbeing	-5
Santiago Atitlán, community 1	*Terapeutas Mayas*	Risk of death	Emotional distress	3
Santiago Atitlán, community 1	*Terapeutas Mayas*	Problems	Emotional distress	5
Santiago Atitlán, community 1	*Terapeutas Mayas*	Problems	Family separation & neglect	5
Santiago Atitlán, community 1	*Terapeutas Mayas*	Substance use	Men's wellbeing	-5
Santiago Atitlán, community 1	*Terapeutas Mayas*	Substance use	Emotional distress	5
Santiago Atitlán, community 1	*Terapeutas Mayas*	Substance use	Poor physical health	5
Santiago Atitlán, community 1	*Terapeutas Mayas*	Substance use	Infidelity	5
Santiago	*Terapeutas*	Suicidality	Men's wellbeing	-5

Atitlán, community 1	*Mayas*			
Santiago Atitlán, community 1	*Terapeutas Mayas*	Infidelity	Family separation & neglect	5
Santiago Atitlán, community 1	*Terapeutas Mayas*	Infidelity	Lack of affectionate, trusting, supportive family relationships	5
Santiago Atitlán, community 1	*Terapeutas Mayas*	Infidelity	Poor physical health	3
Santiago Atitlán, community 1	Young adult men	Emotional distress	Men's wellbeing	-4.5
Santiago Atitlán, community 1	Young adult men	Emotional distress	Misuse of technology	4
Santiago Atitlán, community 1	Young adult men	Emotional distress	Substance use	5
Santiago Atitlán, community 1	Young adult men	Emotional distress	Suicidality	4
Santiago Atitlán, community 1	Young adult men	Social isolation	Emotional distress	5
Santiago Atitlán, community 1	Young adult men	Social isolation	Low self-esteem	3
Santiago Atitlán, community 1	Young adult men	Social isolation	Poor physical health	5
Santiago Atitlán, community 1	Young adult men	Social isolation	Suicidality	5
Santiago Atitlán, community 1	Young adult men	Negative social influences	Personal characteristics that negatively affect social harmony	4
Santiago Atitlán, community 1	Young adult men	Negative social influences	Substance use	5
Santiago Atitlán, community 1	Young adult men	Unemployment	Lack of affectionate, trusting, supportive family relationships	5
Santiago Atitlán,	Young adult men	Unemployment	Irresponsibility	4.5

community 1				
Santiago Atitlán, community 1	Young adult men	Unemployment	Lack of formal education	5
Santiago Atitlán, community 1	Young adult men	Family separation & neglect	Emotional distress	5
Santiago Atitlán, community 1	Young adult men	Family separation & neglect	Social isolation	4
Santiago Atitlán, community 1	Young adult men	Family separation & neglect	Negative social influences	5
Santiago Atitlán, community 1	Young adult men	Family separation & neglect	Irresponsibility	5
Santiago Atitlán, community 1	Young adult men	Family separation & neglect	Low self-esteem	3
Santiago Atitlán, community 1	Young adult men	Lack of affectionate, trusting, supportive family relationships	Social isolation	4
Santiago Atitlán, community 1	Young adult men	Lack of affectionate, trusting, supportive family relationships	Sports/recreation	4
Santiago Atitlán, community 1	Young adult men	Lack of affectionate, trusting, supportive family relationships	Disrupted family education	4.33
Santiago Atitlán, community 1	Young adult men	Lack of affectionate, trusting, supportive family relationships	Substance use	3
Santiago Atitlán, community 1	Young adult men	Irresponsibility	Negative social influences	5
Santiago Atitlán, community 1	Young adult men	Irresponsibility	Unemployment	5
Santiago Atitlán, community 1	Young adult men	Irresponsibility	Substance use	4
Santiago Atitlán, community 1	Young adult men	Bad thoughts	Early dating/marriage/sex/pregnancy	5
Santiago Atitlán, community 1	Young adult men	Bad thoughts	Poor physical health	5

Santiago Atitlán, community 1	Young adult men	Bad thoughts	Infidelity	5
Santiago Atitlán, community 1	Young adult men	Personal characteristics that negatively affect social harmony	Lack of affectionate, trusting, supportive family relationships	5
Santiago Atitlán, community 1	Young adult men	Personal characteristics that negatively affect social harmony	Disrupted family education	5
Santiago Atitlán, community 1	Young adult men	Personal characteristics that negatively affect social harmony	Misuse of technology	5
Santiago Atitlán, community 1	Young adult men	Personal characteristics that negatively affect social harmony	Infidelity	5
Santiago Atitlán, community 1	Young adult men	Sports/recreation	Emotional distress	-2
Santiago Atitlán, community 1	Young adult men	Sports/recreation	Social isolation	-4
Santiago Atitlán, community 1	Young adult men	Sports/recreation	Irresponsibility	3
Santiago Atitlán, community 1	Young adult men	Lack of formal education	Unemployment	5
Santiago Atitlán, community 1	Young adult men	Lack of formal education	Misuse of technology	5
Santiago Atitlán, community 1	Young adult men	Disrupted family education	Negative social influences	5
Santiago Atitlán, community 1	Young adult men	Disrupted family education	Unemployment	5
Santiago Atitlán, community 1	Young adult men	Disrupted family education	Irresponsibility	5
Santiago Atitlán, community 1	Young adult men	Disrupted family education	Personal characteristics that negatively affect social harmony	5
Santiago Atitlán, community 1	Young adult men	Disrupted family education	Disrupted family education	5
Santiago	Young adult	Disrupted family	Poor physical health	4

Atitlán, community 1	men	education		
Santiago Atitlán, community 1	Young adult men	Low self-esteem	Emotional distress	4
Santiago Atitlán, community 1	Young adult men	Low self-esteem	Lack of affectionate, trusting, supportive family relationships	3
Santiago Atitlán, community 1	Young adult men	Low self-esteem	Misuse of technology	5
Santiago Atitlán, community 1	Young adult men	Misuse of technology	Bad thoughts	5
Santiago Atitlán, community 1	Young adult men	Misuse of technology	Poor physical health	5
Santiago Atitlán, community 1	Young adult men	Misuse of technology	Substance use	5
Santiago Atitlán, community 1	Young adult men	Misuse of technology	Infidelity	4
Santiago Atitlán, community 1	Young adult men	Poor physical health	Men's wellbeing	-3
Santiago Atitlán, community 1	Young adult men	Risk of death	Men's wellbeing	-5
Santiago Atitlán, community 1	Young adult men	Substance use	Men's wellbeing	-5
Santiago Atitlán, community 1	Young adult men	Substance use	Emotional distress	5
Santiago Atitlán, community 1	Young adult men	Substance use	Family separation & neglect	5
Santiago Atitlán, community 1	Young adult men	Substance use	Risk of death	5
Santiago Atitlán, community 1	Young adult men	Substance use	Suicidality	5
Santiago Atitlán,	Young adult men	Suicidality	Men's wellbeing	-5

community 1				
Santiago Atitlán, community 1	Young adult men	Infidelity	Social isolation	5
Santiago Atitlán, community 1	Young adult men	Infidelity	Early dating/marriage/sex/pregnancy	5
Santiago Atitlán, community 1	Young adult men	Infidelity	Personal characteristics that negatively affect social harmony	5
Santiago Atitlán, community 1	Young adult men	Infidelity	Misuse of technology	5
Santiago Atitlán, community 1	Young adult men	Infidelity	Substance use	5
Santiago Atitlán, community 1	Young adult men	Infidelity	Suicidality	5
Santiago Atitlán, community 1	Young adult women	Emotional distress	Men's wellbeing	-5
Santiago Atitlán, community 1	Young adult women	Emotional distress	Family separation & neglect	3
Santiago Atitlán, community 1	Young adult women	Emotional distress	Poor physical health	3.5
Santiago Atitlán, community 1	Young adult women	Lack of access to health services and health information	Unwanted pregnancies	4
Santiago Atitlán, community 1	Young adult women	Social isolation	Lack of access to health services and health information	5
Santiago Atitlán, community 1	Young adult women	Social isolation	Theft	3
Santiago Atitlán, community 1	Young adult women	Social isolation	Risk of death	5
Santiago Atitlán, community 1	Young adult women	Social isolation	Substance use	3
Santiago Atitlán, community 1	Young adult women	Social isolation	Suicidality	5

Santiago Atitlán, community 1	Young adult women	Negative social influences	Irresponsibility	5
Santiago Atitlán, community 1	Young adult women	Negative social influences	Substance use	4
Santiago Atitlán, community 1	Young adult women	Witchcraft	Family separation & neglect	5
Santiago Atitlán, community 1	Young adult women	Witchcraft	Personal characteristics that negatively affect social harmony	5
Santiago Atitlán, community 1	Young adult women	Witchcraft	Lack of religious faith	5
Santiago Atitlán, community 1	Young adult women	Witchcraft	Poor physical health	5
Santiago Atitlán, community 1	Young adult women	Witchcraft	Risk of death	5
Santiago Atitlán, community 1	Young adult women	Not communicating feelings/ seeking support	Men's wellbeing	-3
Santiago Atitlán, community 1	Young adult women	Not communicating feelings/ seeking support	Social isolation	5
Santiago Atitlán, community 1	Young adult women	Not communicating feelings/ seeking support	Not respecting customs	3
Santiago Atitlán, community 1	Young adult women	Not communicating feelings/ seeking support	Substance use	3
Santiago Atitlán, community 1	Young adult women	Forced marriage	Unwanted pregnancies	5
Santiago Atitlán, community 1	Young adult women	Forced marriage	Misuse of technology	5
Santiago Atitlán, community 1	Young adult women	Forced marriage	Substance use	5
Santiago Atitlán, community 1	Young adult women	Forced marriage	Infidelity	5
Santiago	Young adult	Basic resource	Irresponsibility	5

Atitlán, community 1	women	insecurity		
Santiago Atitlán, community 1	Young adult women	Basic resource insecurity	Poor health promotive care practices	5
Santiago Atitlán, community 1	Young adult women	Unemployment	Basic resource insecurity	5
Santiago Atitlán, community 1	Young adult women	Unemployment	Child labor	5
Santiago Atitlán, community 1	Young adult women	Unemployment	Irresponsibility	5
Santiago Atitlán, community 1	Young adult women	Unemployment	Theft	5
Santiago Atitlán, community 1	Young adult women	Unemployment	Suicidality	3
Santiago Atitlán, community 1	Young adult women	Child labor	Poor physical health	3
Santiago Atitlán, community 1	Young adult women	Family separation & neglect	Substance use	5
Santiago Atitlán, community 1	Young adult women	Domestic violence	Emotional distress	5
Santiago Atitlán, community 1	Young adult women	Domestic violence	Family separation & neglect	5
Santiago Atitlán, community 1	Young adult women	Domestic violence	Unwanted pregnancies	4
Santiago Atitlán, community 1	Young adult women	Domestic violence	Low self-esteem	3
Santiago Atitlán, community 1	Young adult women	Domestic violence	Poor physical health	3
Santiago Atitlán, community 1	Young adult women	Domestic violence	Substance use	5
Santiago Atitlán,	Young adult women	Domestic violence	Infidelity	3

community 1				
Santiago Atitlán, community 1	Young adult women	Lack of affectionate, trusting, supportive family relationships	Family separation & neglect	3
Santiago Atitlán, community 1	Young adult women	Lack of affectionate, trusting, supportive family relationships	Harmful gender norms	4.5
Santiago Atitlán, community 1	Young adult women	Lack of affectionate, trusting, supportive family relationships	Personal characteristics that negatively affect social harmony	3
Santiago Atitlán, community 1	Young adult women	Lack of affectionate, trusting, supportive family relationships	Infidelity	5
Santiago Atitlán, community 1	Young adult women	Unequal power relationship in couple	Family separation & neglect	3
Santiago Atitlán, community 1	Young adult women	Harmful gender norms	Domestic violence	4
Santiago Atitlán, community 1	Young adult women	Harmful gender norms	Low self-esteem	4
Santiago Atitlán, community 1	Young adult women	Irresponsibility	Basic resource insecurity	5
Santiago Atitlán, community 1	Young adult women	Irresponsibility	Unemployment	3
Santiago Atitlán, community 1	Young adult women	Irresponsibility	Bad thoughts	5
Santiago Atitlán, community 1	Young adult women	Irresponsibility	Infidelity	5
Santiago Atitlán, community 1	Young adult women	Theft	Prison	5
Santiago Atitlán, community 1	Young adult women	Prison	Family separation & neglect	3
Santiago Atitlán, community 1	Young adult women	Bad thoughts	Emotional distress	5
Santiago Atitlán, community 1	Young adult women	Bad thoughts	Irresponsibility	4

Santiago Atitlán, community 1	Young adult women	Personal characteristics that negatively affect social harmony	Negative social influences	5
Santiago Atitlán, community 1	Young adult women	Personal characteristics that negatively affect social harmony	Forced marriage	5
Santiago Atitlán, community 1	Young adult women	Personal characteristics that negatively affect social harmony	Domestic violence	5
Santiago Atitlán, community 1	Young adult women	Personal characteristics that negatively affect social harmony	Lack of affectionate, trusting, supportive family relationships	5
Santiago Atitlán, community 1	Young adult women	Personal characteristics that negatively affect social harmony	Unequal power relationship in couple	3
Santiago Atitlán, community 1	Young adult women	Personal characteristics that negatively affect social harmony	Infidelity	3
Santiago Atitlán, community 1	Young adult women	Poor health promotive care practices	Poor physical health	4
Santiago Atitlán, community 1	Young adult women	Unwanted pregnancies	Basic resource insecurity	4
Santiago Atitlán, community 1	Young adult women	Unwanted pregnancies	Child labor	3
Santiago Atitlán, community 1	Young adult women	Lack of religious faith	Witchcraft	5
Santiago Atitlán, community 1	Young adult women	Lack of religious faith	Not communicating feelings/ seeking support	5
Santiago Atitlán, community 1	Young adult women	Lack of religious faith	Poor physical health	5
Santiago Atitlán, community 1	Young adult women	Lack of religious faith	Bars	5
Santiago Atitlán, community 1	Young adult women	Lack of religious faith	Infidelity	5
Santiago Atitlán, community 1	Young adult women	Not respecting customs	Men's wellbeing	-4
Santiago	Young adult	Not respecting customs	Poor physical health	5

Atitlán, community 1	women			
Santiago Atitlán, community 1	Young adult women	Low self-esteem	Not communicating feelings/ seeking support	5
Santiago Atitlán, community 1	Young adult women	Misuse of technology	Infidelity	5
Santiago Atitlán, community 1	Young adult women	Poor physical health	Men's wellbeing	-4.67
Santiago Atitlán, community 1	Young adult women	Risk of death	Men's wellbeing	-5
Santiago Atitlán, community 1	Young adult women	Substance use	Men's wellbeing	-5
Santiago Atitlán, community 1	Young adult women	Substance use	Emotional distress	4.5
Santiago Atitlán, community 1	Young adult women	Substance use	Social isolation	5
Santiago Atitlán, community 1	Young adult women	Substance use	Domestic violence	4.33
Santiago Atitlán, community 1	Young adult women	Substance use	Lack of affectionate, trusting, supportive family relationships	5
Santiago Atitlán, community 1	Young adult women	Substance use	Lack of religious faith	5
Santiago Atitlán, community 1	Young adult women	Substance use	Poor physical health	4.5
Santiago Atitlán, community 1	Young adult women	Substance use	Risk of death	5
Santiago Atitlán, community 1	Young adult women	Bars	Irresponsibility	5
Santiago Atitlán, community 1	Young adult women	Bars	Lack of religious faith	5
Santiago Atitlán,	Young adult women	Bars	Substance use	5

community 1				
Santiago Atitlán, community 1	Young adult women	Suicidality	Men's wellbeing	-5
Santiago Atitlán, community 1	Young adult women	Infidelity	Family separation & neglect	5
Santiago Atitlán, community 1	Young adult women	Infidelity	Domestic violence	5
Santiago Atitlán, community 1	Young adult women	Infidelity	Lack of affectionate, trusting, supportive family relationships	5
Santiago Atitlán, community 1	Young adult women	Infidelity	Lack of religious faith	5
Santiago Atitlán, community 1	Young adult women	Infidelity	Poor physical health	5
Santiago Atitlán, community 1	Young adult women	Infidelity	Substance use	5
Santiago Atitlán, community 1	Older adult men	Emotional distress	Men's wellbeing	-4
Santiago Atitlán, community 1	Older adult men	Emotional distress	Poor physical health	4
Santiago Atitlán, community 1	Older adult men	Emotional distress	Substance use	2.5
Santiago Atitlán, community 1	Older adult men	Emotional distress	Suicidality	5
Santiago Atitlán, community 1	Older adult men	Lack of access to health services and health information	Risk of death	5
Santiago Atitlán, community 1	Older adult men	Negative social influences	Emotional distress	2
Santiago Atitlán, community 1	Older adult men	Negative social influences	Family separation & neglect	4
Santiago Atitlán, community 1	Older adult men	Negative social influences	Irresponsibility	4

Santiago Atitlán, community 1	Older adult men	Negative social influences	Problems	2
Santiago Atitlán, community 1	Older adult men	Witchcraft	Men's wellbeing	-5
Santiago Atitlán, community 1	Older adult men	Basic resource insecurity	Emotional distress	3
Santiago Atitlán, community 1	Older adult men	Basic resource insecurity	Lack of access to health services and health information	5
Santiago Atitlán, community 1	Older adult men	Basic resource insecurity	Theft	4
Santiago Atitlán, community 1	Older adult men	Basic resource insecurity	Poor physical health	5
Santiago Atitlán, community 1	Older adult men	Family separation & neglect	Lack of affectionate, trusting, supportive family relationships	4
Santiago Atitlán, community 1	Older adult men	Family separation & neglect	Substance use	3
Santiago Atitlán, community 1	Older adult men	Domestic violence	Poor physical health	5
Santiago Atitlán, community 1	Older adult men	Lack of affectionate, trusting, supportive family relationships	Men's wellbeing	-5
Santiago Atitlán, community 1	Older adult men	Lack of affectionate, trusting, supportive family relationships	Domestic violence	5
Santiago Atitlán, community 1	Older adult men	Lack of affectionate, trusting, supportive family relationships	Problems	3
Santiago Atitlán, community 1	Older adult men	Lack of affectionate, trusting, supportive family relationships	Substance use	4
Santiago Atitlán, community 1	Older adult men	Lack of affectionate, trusting, supportive family relationships	Infidelity	4.25
Santiago Atitlán, community 1	Older adult men	Harmful gender norms	Emotional distress	2
Santiago	Older adult	Harmful gender norms	Lack of affectionate,	4

Atitlán, community 1	men		trusting, supportive family relationships	
Santiago Atitlán, community 1	Older adult men	Harmful gender norms	Problems	3
Santiago Atitlán, community 1	Older adult men	Irresponsibility	Men's wellbeing	-3
Santiago Atitlán, community 1	Older adult men	Irresponsibility	Emotional distress	3
Santiago Atitlán, community 1	Older adult men	Irresponsibility	Theft	5
Santiago Atitlán, community 1	Older adult men	Theft	Basic resource insecurity	5
Santiago Atitlán, community 1	Older adult men	Theft	Risk of death	4
Santiago Atitlán, community 1	Older adult men	Personal characteristics that negatively affect social harmony	Negative social influences	3
Santiago Atitlán, community 1	Older adult men	Personal characteristics that negatively affect social harmony	Lack of affectionate, trusting, supportive family relationships	3
Santiago Atitlán, community 1	Older adult men	Personal characteristics that negatively affect social harmony	Problems	5
Santiago Atitlán, community 1	Older adult men	Personal characteristics that negatively affect social harmony	Substance use	3
Santiago Atitlán, community 1	Older adult men	Lack of religious faith	Men's wellbeing	-5
Santiago Atitlán, community 1	Older adult men	Lack of religious faith	Emotional distress	5
Santiago Atitlán, community 1	Older adult men	Not respecting customs	Men's wellbeing	-5
Santiago Atitlán, community 1	Older adult men	Not respecting customs	Emotional distress	4.5
Santiago Atitlán,	Older adult men	Not respecting customs	Basic resource insecurity	2

community 1				
Santiago Atitlán, community 1	Older adult men	Not respecting customs	Irresponsibility	4
Santiago Atitlán, community 1	Older adult men	Not respecting customs	Poor physical health	3.8
Santiago Atitlán, community 1	Older adult men	Not respecting customs	Infertility	5
Santiago Atitlán, community 1	Older adult men	Poor physical health	Men's wellbeing	-5
Santiago Atitlán, community 1	Older adult men	Infertility	Poor physical health	3
Santiago Atitlán, community 1	Older adult men	Risk of death	Men's wellbeing	-5
Santiago Atitlán, community 1	Older adult men	Problems	Men's wellbeing	-5
Santiago Atitlán, community 1	Older adult men	Problems	Basic resource insecurity	3
Santiago Atitlán, community 1	Older adult men	Problems	Substance use	4
Santiago Atitlán, community 1	Older adult men	Substance use	Men's wellbeing	-5
Santiago Atitlán, community 1	Older adult men	Substance use	Family separation & neglect	5
Santiago Atitlán, community 1	Older adult men	Substance use	Domestic violence	1
Santiago Atitlán, community 1	Older adult men	Substance use	Infidelity	5
Santiago Atitlán, community 1	Older adult men	Suicidality	Men's wellbeing	-5
Santiago Atitlán, community 1	Older adult men	Suicidality	Basic resource insecurity	5

Santiago Atitlán, community 1	Older adult men	Infidelity	Witchcraft	5
Santiago Atitlán, community 1	Older adult men	Infidelity	Family separation & neglect	5
Santiago Atitlán, community 1	Older adult men	Infidelity	Lack of affectionate, trusting, supportive family relationships	4
Santiago Atitlán, community 1	Older adult men	Infidelity	Risk of death	5
Santiago Atitlán, community 1	Older adult men	Infidelity	Problems	5
Santiago Atitlán, community 1	Older adult women	Emotional distress	Men's wellbeing	-4.33
Santiago Atitlán, community 1	Older adult women	Emotional distress	Family separation & neglect	4
Santiago Atitlán, community 1	Older adult women	Emotional distress	Poor physical health	3
Santiago Atitlán, community 1	Older adult women	Emotional distress	Risk of death	5
Santiago Atitlán, community 1	Older adult women	Emotional distress	Infidelity	5
Santiago Atitlán, community 1	Older adult women	Negative social influences	Personal characteristics that negatively affect social harmony	5
Santiago Atitlán, community 1	Older adult women	Negative social influences	Substance use	5
Santiago Atitlán, community 1	Older adult women	Not communicating feelings/ seeking support	Poor physical health	4
Santiago Atitlán, community 1	Older adult women	Not communicating feelings/ seeking support	Substance use	5
Santiago Atitlán, community 1	Older adult women	Basic resource insecurity	Family separation & neglect	4
Santiago	Older adult	Basic resource	Theft	5

Atitlán, community 1	women	insecurity		
Santiago Atitlán, community 1	Older adult women	Basic resource insecurity	Poor health promotive care practices	3
Santiago Atitlán, community 1	Older adult women	Basic resource insecurity	Lack of formal education	5
Santiago Atitlán, community 1	Older adult women	Basic resource insecurity	Substance use	5
Santiago Atitlán, community 1	Older adult women	Unemployment	Basic resource insecurity	4
Santiago Atitlán, community 1	Older adult women	Unemployment	Lack of formal education	5
Santiago Atitlán, community 1	Older adult women	Unemployment	Substance use	5
Santiago Atitlán, community 1	Older adult women	Family separation & neglect	Basic resource insecurity	3
Santiago Atitlán, community 1	Older adult women	Family separation & neglect	Personal characteristics that negatively affect social harmony	5
Santiago Atitlán, community 1	Older adult women	Family separation & neglect	Substance use	5
Santiago Atitlán, community 1	Older adult women	Lack of affectionate, trusting, supportive family relationships	Family separation & neglect	5
Santiago Atitlán, community 1	Older adult women	Lack of affectionate, trusting, supportive family relationships	Low self-esteem	5
Santiago Atitlán, community 1	Older adult women	Irresponsibility	Basic resource insecurity	4.5
Santiago Atitlán, community 1	Older adult women	Irresponsibility	Lack of affectionate, trusting, supportive family relationships	4
Santiago Atitlán, community 1	Older adult women	Irresponsibility	Poor health promotive care practices	3
Santiago Atitlán,	Older adult women	Theft	Risk of death	5

community 1				
Santiago Atitlán, community 1	Older adult women	Personal characteristics that negatively affect social harmony	Not communicating feelings/ seeking support	5
Santiago Atitlán, community 1	Older adult women	Personal characteristics that negatively affect social harmony	Disrupted family education	4.75
Santiago Atitlán, community 1	Older adult women	Personal characteristics that negatively affect social harmony	Substance use	5
Santiago Atitlán, community 1	Older adult women	Personal characteristics that negatively affect social harmony	Infidelity	5
Santiago Atitlán, community 1	Older adult women	Poor health promotive care practices	Poor physical health	3
Santiago Atitlán, community 1	Older adult women	Unwanted pregnancies	Basic resource insecurity	5
Santiago Atitlán, community 1	Older adult women	Unwanted pregnancies	Lack of affectionate, trusting, supportive family relationships	5
Santiago Atitlán, community 1	Older adult women	Lack of formal education	Unemployment	3
Santiago Atitlán, community 1	Older adult women	Lack of formal education	Personal characteristics that negatively affect social harmony	5
Santiago Atitlán, community 1	Older adult women	Lack of formal education	Unwanted pregnancies	5
Santiago Atitlán, community 1	Older adult women	Lack of religious faith	Emotional distress	4
Santiago Atitlán, community 1	Older adult women	Lack of religious faith	Personal characteristics that negatively affect social harmony	5
Santiago Atitlán, community 1	Older adult women	Lack of religious faith	Risk of death	5
Santiago Atitlán, community 1	Older adult women	Disrupted family education	Men's wellbeing	-5
Santiago Atitlán, community 1	Older adult women	Disrupted family education	Negative social influences	5

Santiago Atitlán, community 1	Older adult women	Disrupted family education	Personal characteristics that negatively affect social harmony	5
Santiago Atitlán, community 1	Older adult women	Disrupted family education	Lack of religious faith	5
Santiago Atitlán, community 1	Older adult women	Disrupted family education	Infidelity	5
Santiago Atitlán, community 1	Older adult women	Low self-esteem	Not communicating feelings/ seeking support	4
Santiago Atitlán, community 1	Older adult women	Low self-esteem	Personal characteristics that negatively affect social harmony	4.5
Santiago Atitlán, community 1	Older adult women	Misuse of technology	Emotional distress	3
Santiago Atitlán, community 1	Older adult women	Misuse of technology	Irresponsibility	5
Santiago Atitlán, community 1	Older adult women	Misuse of technology	Low self-esteem	5
Santiago Atitlán, community 1	Older adult women	Poor physical health	Men's wellbeing	-3
Santiago Atitlán, community 1	Older adult women	Poor physical health	Basic resource insecurity	3
Santiago Atitlán, community 1	Older adult women	Poor physical health	Risk of death	3
Santiago Atitlán, community 1	Older adult women	Risk of death	Men's wellbeing	-5
Santiago Atitlán, community 1	Older adult women	Risk of death	Family separation & neglect	5
Santiago Atitlán, community 1	Older adult women	Substance use	Men's wellbeing	-4.33
Santiago Atitlán, community 1	Older adult women	Substance use	Emotional distress	4
Santiago	Older adult	Substance use	Basic resource	5

Atitlán, community 1	women		insecurity	
Santiago Atitlán, community 1	Older adult women	Substance use	Theft	5
Santiago Atitlán, community 1	Older adult women	Substance use	Poor health promotive care practices	4
Santiago Atitlán, community 1	Older adult women	Substance use	Poor physical health	4.33
Santiago Atitlán, community 1	Older adult women	Substance use	Risk of death	4.33
Santiago Atitlán, community 1	Older adult women	Infidelity	Family separation & neglect	5
Santiago Atitlán, community 1	Older adult women	Infidelity	Misuse of technology	5
Santiago Atitlán, community 1	Older adult women	Infidelity	Poor physical health	5
Santiago Atitlán, community 2	Adult men	Emotional distress	Men's wellbeing	-5
Santiago Atitlán, community 2	Adult men	Emotional distress	Lack of affectionate, trusting, supportive family relationships	3
Santiago Atitlán, community 2	Adult men	Emotional distress	Personal characteristics that negatively affect social harmony	5
Santiago Atitlán, community 2	Adult men	Social isolation	Low self-esteem	5
Santiago Atitlán, community 2	Adult men	Negative social influences	Irresponsibility	5
Santiago Atitlán, community 2	Adult men	Basic resource insecurity	Men's wellbeing	-3
Santiago Atitlán, community 2	Adult men	Basic resource insecurity	Social isolation	5
Santiago Atitlán,	Adult men	Basic resource insecurity	Unemployment	5

community 2				
Santiago Atitlán, community 2	Adult men	Basic resource insecurity	Theft	5
Santiago Atitlán, community 2	Adult men	Basic resource insecurity	Excessive workload	4
Santiago Atitlán, community 2	Adult men	Basic resource insecurity	Bars	5
Santiago Atitlán, community 2	Adult men	Basic resource insecurity	Infidelity	4
Santiago Atitlán, community 2	Adult men	Unemployment	Basic resource insecurity	5
Santiago Atitlán, community 2	Adult men	Unemployment	Low self-esteem	3
Santiago Atitlán, community 2	Adult men	Family separation & neglect	Lack of affectionate, trusting, supportive family relationships	5
Santiago Atitlán, community 2	Adult men	Family separation & neglect	Misuse of technology	5
Santiago Atitlán, community 2	Adult men	Family separation & neglect	Substance use	5
Santiago Atitlán, community 2	Adult men	Domestic violence	Men's wellbeing	-3
Santiago Atitlán, community 2	Adult men	Lack of affectionate, trusting, supportive family relationships	Emotional distress	5
Santiago Atitlán, community 2	Adult men	Lack of affectionate, trusting, supportive family relationships	Family separation & neglect	5
Santiago Atitlán, community 2	Adult men	Lack of affectionate, trusting, supportive family relationships	Domestic violence	4
Santiago Atitlán, community 2	Adult men	Lack of affectionate, trusting, supportive family relationships	Personal characteristics that negatively affect social harmony	4
Santiago Atitlán, community 2	Adult men	Lack of affectionate, trusting, supportive family relationships	Poor physical health	4

Santiago Atitlán, community 2	Adult men	Lack of affectionate, trusting, supportive family relationships	Substance use	3
Santiago Atitlán, community 2	Adult men	Lack of affectionate, trusting, supportive family relationships	Infidelity	3
Santiago Atitlán, community 2	Adult men	Irresponsibility	Low self-esteem	3
Santiago Atitlán, community 2	Adult men	Theft	Lack of affectionate, trusting, supportive family relationships	3
Santiago Atitlán, community 2	Adult men	Personal characteristics that negatively affect social harmony	Emotional distress	5
Santiago Atitlán, community 2	Adult men	Personal characteristics that negatively affect social harmony	Negative social influences	5
Santiago Atitlán, community 2	Adult men	Personal characteristics that negatively affect social harmony	Domestic violence	2
Santiago Atitlán, community 2	Adult men	Personal characteristics that negatively affect social harmony	Lack of affectionate, trusting, supportive family relationships	4.5
Santiago Atitlán, community 2	Adult men	Personal characteristics that negatively affect social harmony	Irresponsibility	5
Santiago Atitlán, community 2	Adult men	Personal characteristics that negatively affect social harmony	Lack of religious faith	3
Santiago Atitlán, community 2	Adult men	Personal characteristics that negatively affect social harmony	Misuse of technology	3
Santiago Atitlán, community 2	Adult men	Personal characteristics that negatively affect social harmony	Risk of death	3
Santiago Atitlán, community 2	Adult men	Personal characteristics that negatively affect social harmony	Problems	3
Santiago Atitlán, community 2	Adult men	Personal characteristics that negatively affect social harmony	Substance use	3
Santiago Atitlán, community 2	Adult men	Excessive workload	Family separation & neglect	3
Santiago	Adult men	Excessive workload	Poor physical health	4

Atitlán, community 2				
Santiago Atitlán, community 2	Adult men	Lack of religious faith	Men's wellbeing	-5
Santiago Atitlán, community 2	Adult men	Lack of religious faith	Emotional distress	4
Santiago Atitlán, community 2	Adult men	Lack of religious faith	Personal characteristics that negatively affect social harmony	3
Santiago Atitlán, community 2	Adult men	Low self-esteem	Emotional distress	4
Santiago Atitlán, community 2	Adult men	Misuse of technology	Family separation & neglect	5
Santiago Atitlán, community 2	Adult men	Misuse of technology	Personal characteristics that negatively affect social harmony	3
Santiago Atitlán, community 2	Adult men	Misuse of technology	Problems	5
Santiago Atitlán, community 2	Adult men	Misuse of technology	Infidelity	2
Santiago Atitlán, community 2	Adult men	Poor physical health	Men's wellbeing	-5
Santiago Atitlán, community 2	Adult men	Risk of death	Men's wellbeing	-1
Santiago Atitlán, community 2	Adult men	Problems	Men's wellbeing	-5
Santiago Atitlán, community 2	Adult men	Problems	Poor physical health	4
Santiago Atitlán, community 2	Adult men	Substance use	Men's wellbeing	-5
Santiago Atitlán, community 2	Adult men	Substance use	Family separation & neglect	5
Santiago Atitlán,	Adult men	Substance use	Theft	4

community 2				
Santiago Atitlán, community 2	Adult men	Substance use	Infidelity	5
Santiago Atitlán, community 2	Adult men	Bars	Substance use	5
Santiago Atitlán, community 2	Adult men	Suicidality	Men's wellbeing	-1
Santiago Atitlán, community 2	Adult men	Suicidality	Risk of death	5
Santiago Atitlán, community 2	Adult men	Infidelity	Family separation & neglect	5
Santiago Atitlán, community 2	Adult men	Infidelity	Lack of affectionate, trusting, supportive family relationships	5
Santiago Atitlán, community 2	Adult men	Infidelity	Personal characteristics that negatively affect social harmony	5
Santiago Atitlán, community 2	Adult men	Infidelity	Poor physical health	5
Santiago Atitlán, community 2	Adult men	Infidelity	Problems	5
Santiago Atitlán, community 2	Adult women	Emotional distress	Men's wellbeing	-5
Santiago Atitlán, community 2	Adult women	Emotional distress	Domestic violence	5
Santiago Atitlán, community 2	Adult women	Negative social influences	Emotional distress	5
Santiago Atitlán, community 2	Adult women	Negative social influences	Problems	5
Santiago Atitlán, community 2	Adult women	Negative social influences	Substance use	5
Santiago Atitlán, community 2	Adult women	Not communicating feelings/ seeking support	Men's wellbeing	-5

Santiago Atitlán, community 2	Adult women	Not communicating feelings/ seeking support	Emotional distress	5
Santiago Atitlán, community 2	Adult women	Not communicating feelings/ seeking support	Lack of affectionate, trusting, supportive family relationships	4
Santiago Atitlán, community 2	Adult women	Not communicating feelings/ seeking support	Poor physical health	4
Santiago Atitlán, community 2	Adult women	Not communicating feelings/ seeking support	Substance use	4
Santiago Atitlán, community 2	Adult women	Basic resource insecurity	Emotional distress	5
Santiago Atitlán, community 2	Adult women	Basic resource insecurity	Unequal power relationship in couple	-4
Santiago Atitlán, community 2	Adult women	Basic resource insecurity	Theft	4
Santiago Atitlán, community 2	Adult women	Basic resource insecurity	Poor health promotive care practices	4
Santiago Atitlán, community 2	Adult women	Basic resource insecurity	Infidelity	-5
Santiago Atitlán, community 2	Adult women	Unemployment	Basic resource insecurity	5
Santiago Atitlán, community 2	Adult women	Unemployment	Child labor	5
Santiago Atitlán, community 2	Adult women	Unemployment	Irresponsibility	5
Santiago Atitlán, community 2	Adult women	Unemployment	Theft	5
Santiago Atitlán, community 2	Adult women	Child labor	Emotional distress	4
Santiago Atitlán, community 2	Adult women	Family separation & neglect	Emotional distress	2
Santiago	Adult women	Family separation &	Negative social	5

Atitlán, community 2		neglect	influences	
Santiago Atitlán, community 2	Adult women	Domestic violence	Family separation & neglect	5
Santiago Atitlán, community 2	Adult women	Lack of affectionate, trusting, supportive family relationships	Emotional distress	5
Santiago Atitlán, community 2	Adult women	Lack of affectionate, trusting, supportive family relationships	Negative social influences	3.5
Santiago Atitlán, community 2	Adult women	Lack of affectionate, trusting, supportive family relationships	Not communicating feelings/ seeking support	5
Santiago Atitlán, community 2	Adult women	Lack of affectionate, trusting, supportive family relationships	Family separation & neglect	4
Santiago Atitlán, community 2	Adult women	Lack of affectionate, trusting, supportive family relationships	Bad thoughts	5
Santiago Atitlán, community 2	Adult women	Unequal power relationship in couple	Emotional distress	4
Santiago Atitlán, community 2	Adult women	Unequal power relationship in couple	Lack of affectionate, trusting, supportive family relationships	5
Santiago Atitlán, community 2	Adult women	Harmful gender norms	Emotional distress	5
Santiago Atitlán, community 2	Adult women	Harmful gender norms	Not communicating feelings/ seeking support	5
Santiago Atitlán, community 2	Adult women	Harmful gender norms	Lack of affectionate, trusting, supportive family relationships	5
Santiago Atitlán, community 2	Adult women	Harmful gender norms	Unequal power relationship in couple	5
Santiago Atitlán, community 2	Adult women	Irresponsibility	Basic resource insecurity	5
Santiago Atitlán, community 2	Adult women	Irresponsibility	Unemployment	5
Santiago Atitlán,	Adult women	Irresponsibility	Child labor	5

community 2				
Santiago Atitlán, community 2	Adult women	Theft	Unemployment	5
Santiago Atitlán, community 2	Adult women	Bad thoughts	Emotional distress	4
Santiago Atitlán, community 2	Adult women	Bad thoughts	Unequal power relationship in couple	4
Santiago Atitlán, community 2	Adult women	Personal characteristics that negatively affect social harmony	Men's wellbeing	-5
Santiago Atitlán, community 2	Adult women	Personal characteristics that negatively affect social harmony	Negative social influences	5
Santiago Atitlán, community 2	Adult women	Personal characteristics that negatively affect social harmony	Basic resource insecurity	4
Santiago Atitlán, community 2	Adult women	Personal characteristics that negatively affect social harmony	Lack of affectionate, trusting, supportive family relationships	5
Santiago Atitlán, community 2	Adult women	Personal characteristics that negatively affect social harmony	Bad thoughts	5
Santiago Atitlán, community 2	Adult women	Personal characteristics that negatively affect social harmony	Substance use	5
Santiago Atitlán, community 2	Adult women	Poor health promotive care practices	Poor physical health	4.5
Santiago Atitlán, community 2	Adult women	Poor physical health	Men's wellbeing	-4
Santiago Atitlán, community 2	Adult women	Poor physical health	Risk of death	3
Santiago Atitlán, community 2	Adult women	Risk of death	Men's wellbeing	-5
Santiago Atitlán, community 2	Adult women	Risk of death	Emotional distress	5
Santiago Atitlán, community 2	Adult women	Problems	Substance use	3

Santiago Atitlán, community 2	Adult women	Substance use	Men's wellbeing	-5
Santiago Atitlán, community 2	Adult women	Substance use	Emotional distress	3.5
Santiago Atitlán, community 2	Adult women	Substance use	Domestic violence	3
Santiago Atitlán, community 2	Adult women	Substance use	Theft	5
Santiago Atitlán, community 2	Adult women	Substance use	Poor physical health	4.5
Santiago Atitlán, community 2	Adult women	Infidelity	Basic resource insecurity	5
Santiago Atitlán, community 2	Adult women	Infidelity	Family separation & neglect	5
Santiago Atitlán, community 2	Adult women	Infidelity	Substance use	5
Santiago Atitlán, community 2	*Terapeutas Mayas*	Emotional distress	Men's wellbeing	-5
Santiago Atitlán, community 2	*Terapeutas Mayas*	Emotional distress	Domestic violence	5
Santiago Atitlán, community 2	*Terapeutas Mayas*	Emotional distress	Not sleeping well	5
Santiago Atitlán, community 2	*Terapeutas Mayas*	Emotional distress	Poor physical health	3
Santiago Atitlán, community 2	*Terapeutas Mayas*	Social isolation	Emotional distress	5
Santiago Atitlán, community 2	*Terapeutas Mayas*	Social isolation	Lack of affectionate, trusting, supportive family relationships	5
Santiago Atitlán, community 2	*Terapeutas Mayas*	Negative social influences	Emotional distress	5
Santiago	*Terapeutas*	Negative social	Family separation &	5

Atitlán, community 2	*Mayas*	influences	neglect	
Santiago Atitlán, community 2	*Terapeutas Mayas*	Negative social influences	Bad thoughts	5
Santiago Atitlán, community 2	*Terapeutas Mayas*	Negative social influences	Personal characteristics that negatively affect social harmony	5
Santiago Atitlán, community 2	*Terapeutas Mayas*	Negative social influences	Not respecting customs	5
Santiago Atitlán, community 2	*Terapeutas Mayas*	Basic resource insecurity	Emotional distress	5
Santiago Atitlán, community 2	*Terapeutas Mayas*	Basic resource insecurity	Excessive workload	5
Santiago Atitlán, community 2	*Terapeutas Mayas*	Basic resource insecurity	Poor physical health	5
Santiago Atitlán, community 2	*Terapeutas Mayas*	Family separation & neglect	Emotional distress	5
Santiago Atitlán, community 2	*Terapeutas Mayas*	Family separation & neglect	Infidelity	5
Santiago Atitlán, community 2	*Terapeutas Mayas*	Domestic violence	Emotional distress	5
Santiago Atitlán, community 2	*Terapeutas Mayas*	Lack of affectionate, trusting, supportive family relationships	Emotional distress	5
Santiago Atitlán, community 2	*Terapeutas Mayas*	Lack of affectionate, trusting, supportive family relationships	Unequal power relationship in couple	5
Santiago Atitlán, community 2	*Terapeutas Mayas*	Lack of affectionate, trusting, supportive family relationships	Infidelity	5
Santiago Atitlán, community 2	*Terapeutas Mayas*	Unequal power relationship in couple	Domestic violence	5
Santiago Atitlán, community 2	*Terapeutas Mayas*	Unequal power relationship in couple	Excessive workload	4
Santiago Atitlán,	*Terapeutas Mayas*	Irresponsibility	Basic resource insecurity	5

community 2				
Santiago Atitlán, community 2	*Terapeutas Mayas*	Irresponsibility	Theft	5
Santiago Atitlán, community 2	*Terapeutas Mayas*	Irresponsibility	Poor physical health	5
Santiago Atitlán, community 2	*Terapeutas Mayas*	Theft	Risk of death	5
Santiago Atitlán, community 2	*Terapeutas Mayas*	Bad thoughts	Social isolation	5
Santiago Atitlán, community 2	*Terapeutas Mayas*	Personal characteristics that negatively affect social harmony	Men's wellbeing	-5
Santiago Atitlán, community 2	*Terapeutas Mayas*	Personal characteristics that negatively affect social harmony	Emotional distress	5
Santiago Atitlán, community 2	*Terapeutas Mayas*	Personal characteristics that negatively affect social harmony	Social isolation	5
Santiago Atitlán, community 2	*Terapeutas Mayas*	Personal characteristics that negatively affect social harmony	Negative social influences	5
Santiago Atitlán, community 2	*Terapeutas Mayas*	Personal characteristics that negatively affect social harmony	Irresponsibility	2
Santiago Atitlán, community 2	*Terapeutas Mayas*	Personal characteristics that negatively affect social harmony	Theft	5
Santiago Atitlán, community 2	*Terapeutas Mayas*	Personal characteristics that negatively affect social harmony	Bad thoughts	5
Santiago Atitlán, community 2	*Terapeutas Mayas*	Personal characteristics that negatively affect social harmony	Risk of death	5
Santiago Atitlán, community 2	*Terapeutas Mayas*	Personal characteristics that negatively affect social harmony	Substance use	5
Santiago Atitlán, community 2	*Terapeutas Mayas*	Excessive workload	Social isolation	3
Santiago Atitlán, community 2	*Terapeutas Mayas*	Excessive workload	Poor physical health	3.5

Santiago Atitlán, community 2	*Terapeutas Mayas*	Not sleeping well	Men's wellbeing	-5
Santiago Atitlán, community 2	*Terapeutas Mayas*	Unwanted pregnancies	Men's wellbeing	-5
Santiago Atitlán, community 2	*Terapeutas Mayas*	Unwanted pregnancies	Emotional distress	5
Santiago Atitlán, community 2	*Terapeutas Mayas*	Unwanted pregnancies	Basic resource insecurity	4
Santiago Atitlán, community 2	*Terapeutas Mayas*	Unwanted pregnancies	Family separation & neglect	5
Santiago Atitlán, community 2	*Terapeutas Mayas*	Not respecting customs	Emotional distress	5
Santiago Atitlán, community 2	*Terapeutas Mayas*	Not respecting customs	Family separation & neglect	5
Santiago Atitlán, community 2	*Terapeutas Mayas*	Poor physical health	Men's wellbeing	-5
Santiago Atitlán, community 2	*Terapeutas Mayas*	Risk of death	Men's wellbeing	-5
Santiago Atitlán, community 2	*Terapeutas Mayas*	Substance use	Men's wellbeing	-5
Santiago Atitlán, community 2	*Terapeutas Mayas*	Substance use	Family separation & neglect	5
Santiago Atitlán, community 2	*Terapeutas Mayas*	Substance use	Domestic violence	5
Santiago Atitlán, community 2	*Terapeutas Mayas*	Substance use	Theft	5
Santiago Atitlán, community 2	*Terapeutas Mayas*	Substance use	Personal characteristics that negatively affect social harmony	5
Santiago Atitlán, community 2	*Terapeutas Mayas*	Substance use	Poor physical health	5
Santiago	*Terapeutas*	Substance use	Risk of death	5

Atitlán, community 2	*Mayas*			
Santiago Atitlán, community 2	*Terapeutas Mayas*	Infidelity	Emotional distress	5
Santiago Atitlán, community 2	Young adult men	Emotional distress	Men's wellbeing	-4
Santiago Atitlán, community 2	Young adult men	Emotional distress	Social isolation	4
Santiago Atitlán, community 2	Young adult men	Emotional distress	Domestic violence	5
Santiago Atitlán, community 2	Young adult men	Emotional distress	Substance use	5
Santiago Atitlán, community 2	Young adult men	Social isolation	Emotional distress	4.5
Santiago Atitlán, community 2	Young adult men	Social isolation	Early dating/marriage/sex/pregnancy	5
Santiago Atitlán, community 2	Young adult men	Social isolation	Domestic violence	3
Santiago Atitlán, community 2	Young adult men	Social isolation	Low self-esteem	5
Santiago Atitlán, community 2	Young adult men	Negative social influences	Early dating/marriage/sex/pregnancy	4
Santiago Atitlán, community 2	Young adult men	Negative social influences	Lack of affectionate, trusting, supportive family relationships	4
Santiago Atitlán, community 2	Young adult men	Negative social influences	Irresponsibility	3
Santiago Atitlán, community 2	Young adult men	Negative social influences	Disrupted family education	5
Santiago Atitlán, community 2	Young adult men	Negative social influences	Substance use	4
Santiago Atitlán,	Young adult men	Early dating/marriage/sex/pre	Lack of affectionate, trusting, supportive	5

community 2		gnancy	family relationships	
Santiago Atitlán, community 2	Young adult men	Basic resource insecurity	Social isolation	4
Santiago Atitlán, community 2	Young adult men	Basic resource insecurity	Unemployment	5
Santiago Atitlán, community 2	Young adult men	Basic resource insecurity	Poor health promotive care practices	5
Santiago Atitlán, community 2	Young adult men	Basic resource insecurity	Excessive workload	5
Santiago Atitlán, community 2	Young adult men	Basic resource insecurity	Lack of formal education	5
Santiago Atitlán, community 2	Young adult men	Basic resource insecurity	Bars	5
Santiago Atitlán, community 2	Young adult men	Unemployment	Basic resource insecurity	5
Santiago Atitlán, community 2	Young adult men	Family separation & neglect	Emotional distress	5
Santiago Atitlán, community 2	Young adult men	Family separation & neglect	Social isolation	4
Santiago Atitlán, community 2	Young adult men	Family separation & neglect	Negative social influences	5
Santiago Atitlán, community 2	Young adult men	Family separation & neglect	Irresponsibility	3
Santiago Atitlán, community 2	Young adult men	Domestic violence	Men's wellbeing	-4
Santiago Atitlán, community 2	Young adult men	Domestic violence	Basic resource insecurity	3
Santiago Atitlán, community 2	Young adult men	Domestic violence	Risk of death	4
Santiago Atitlán, community 2	Young adult men	Lack of affectionate, trusting, supportive family relationships	Emotional distress	5

Santiago Atitlán, community 2	Young adult men	Lack of affectionate, trusting, supportive family relationships	Family separation & neglect	4
Santiago Atitlán, community 2	Young adult men	Lack of affectionate, trusting, supportive family relationships	Substance use	3
Santiago Atitlán, community 2	Young adult men	Irresponsibility	Emotional distress	5
Santiago Atitlán, community 2	Young adult men	Irresponsibility	Negative social influences	5
Santiago Atitlán, community 2	Young adult men	Irresponsibility	Lack of affectionate, trusting, supportive family relationships	5
Santiago Atitlán, community 2	Young adult men	Irresponsibility	Poor physical health	3
Santiago Atitlán, community 2	Young adult men	Poor health promotive care practices	Men's wellbeing	-3
Santiago Atitlán, community 2	Young adult men	Poor health promotive care practices	Poor physical health	5
Santiago Atitlán, community 2	Young adult men	Excessive workload	Family separation & neglect	4
Santiago Atitlán, community 2	Young adult men	Excessive workload	Poor physical health	5
Santiago Atitlán, community 2	Young adult men	Lack of formal education	Social isolation	5
Santiago Atitlán, community 2	Young adult men	Lack of formal education	Unemployment	5
Santiago Atitlán, community 2	Young adult men	Lack of formal education	Bars	5
Santiago Atitlán, community 2	Young adult men	Disrupted family education	Negative social influences	5
Santiago Atitlán, community 2	Young adult men	Disrupted family education	Lack of affectionate, trusting, supportive family relationships	5
Santiago	Young adult	Disrupted family	Irresponsibility	4

Atitlán, community 2	men	education		
Santiago Atitlán, community 2	Young adult men	Low self-esteem	Emotional distress	4
Santiago Atitlán, community 2	Young adult men	Poor physical health	Men's wellbeing	-4
Santiago Atitlán, community 2	Young adult men	Poor physical health	Emotional distress	5
Santiago Atitlán, community 2	Young adult men	Risk of death	Men's wellbeing	-5
Santiago Atitlán, community 2	Young adult men	Risk of death	Emotional distress	5
Santiago Atitlán, community 2	Young adult men	Risk of death	Social isolation	3
Santiago Atitlán, community 2	Young adult men	Substance use	Men's wellbeing	-5
Santiago Atitlán, community 2	Young adult men	Substance use	Negative social influences	5
Santiago Atitlán, community 2	Young adult men	Substance use	Basic resource insecurity	5
Santiago Atitlán, community 2	Young adult men	Substance use	Family separation & neglect	5
Santiago Atitlán, community 2	Young adult men	Substance use	Domestic violence	4.5
Santiago Atitlán, community 2	Young adult men	Substance use	Lack of affectionate, trusting, supportive family relationships	5
Santiago Atitlán, community 2	Young adult men	Substance use	Poor physical health	5
Santiago Atitlán, community 2	Young adult men	Substance use	Risk of death	5
Santiago Atitlán,	Young adult men	Bars	Domestic violence	5

community 2				
Santiago Atitlán, community 2	Young adult men	Bars	Poor physical health	3
Santiago Atitlán, community 2	Young adult men	Bars	Substance use	5
Santiago Atitlán, community 2	Young adult women	Emotional distress	Men's wellbeing	-5
Santiago Atitlán, community 2	Young adult women	Emotional distress	Risk of death	3
Santiago Atitlán, community 2	Young adult women	Social isolation	Lack of affectionate, trusting, supportive family relationships	5
Santiago Atitlán, community 2	Young adult women	Social isolation	Low self-esteem	5
Santiago Atitlán, community 2	Young adult women	Negative social influences	Domestic violence	5
Santiago Atitlán, community 2	Young adult women	Negative social influences	Theft	5
Santiago Atitlán, community 2	Young adult women	Negative social influences	Lack of religious faith	5
Santiago Atitlán, community 2	Young adult women	Negative social influences	Substance use	5
Santiago Atitlán, community 2	Young adult women	Not communicating feelings/ seeking support	Emotional distress	4.5
Santiago Atitlán, community 2	Young adult women	Not communicating feelings/ seeking support	Negative social influences	4
Santiago Atitlán, community 2	Young adult women	Not communicating feelings/ seeking support	Substance use	4.33
Santiago Atitlán, community 2	Young adult women	Forced marriage	Family separation & neglect	5
Santiago Atitlán, community 2	Young adult women	Forced marriage	Unwanted pregnancies	4

Santiago Atitlán, community 2	Young adult women	Forced marriage	Disrupted family education	5
Santiago Atitlán, community 2	Young adult women	Forced marriage	Substance use	5
Santiago Atitlán, community 2	Young adult women	Forced marriage	Infidelity	5
Santiago Atitlán, community 2	Young adult women	Basic resource insecurity	Negative social influences	5
Santiago Atitlán, community 2	Young adult women	Basic resource insecurity	Irresponsibility	5
Santiago Atitlán, community 2	Young adult women	Basic resource insecurity	Theft	3
Santiago Atitlán, community 2	Young adult women	Basic resource insecurity	Poor health promotive care practices	5
Santiago Atitlán, community 2	Young adult women	Basic resource insecurity	Lack of formal education	4
Santiago Atitlán, community 2	Young adult women	Basic resource insecurity	Low self-esteem	4
Santiago Atitlán, community 2	Young adult women	Family separation & neglect	Substance use	5
Santiago Atitlán, community 2	Young adult women	Domestic violence	Emotional distress	5
Santiago Atitlán, community 2	Young adult women	Domestic violence	Family separation & neglect	5
Santiago Atitlán, community 2	Young adult women	Lack of affectionate, trusting, supportive family relationships	Emotional distress	4
Santiago Atitlán, community 2	Young adult women	Lack of affectionate, trusting, supportive family relationships	Family separation & neglect	4
Santiago Atitlán, community 2	Young adult women	Lack of affectionate, trusting, supportive family relationships	Harmful gender norms	5
Santiago	Young adult	Harmful gender norms	Emotional distress	5

Atitlán, community 2	women			
Santiago Atitlán, community 2	Young adult women	Harmful gender norms	Social isolation	4
Santiago Atitlán, community 2	Young adult women	Harmful gender norms	Forced marriage	5
Santiago Atitlán, community 2	Young adult women	Harmful gender norms	Family separation & neglect	4
Santiago Atitlán, community 2	Young adult women	Harmful gender norms	Low self-esteem	5
Santiago Atitlán, community 2	Young adult women	Irresponsibility	Negative social influences	4.5
Santiago Atitlán, community 2	Young adult women	Irresponsibility	Basic resource insecurity	5
Santiago Atitlán, community 2	Young adult women	Irresponsibility	Theft	4
Santiago Atitlán, community 2	Young adult women	Irresponsibility	Risk of death	5
Santiago Atitlán, community 2	Young adult women	Theft	Risk of death	5
Santiago Atitlán, community 2	Young adult women	Bad thoughts	Negative social influences	4
Santiago Atitlán, community 2	Young adult women	Bad thoughts	Domestic violence	5
Santiago Atitlán, community 2	Young adult women	Bad thoughts	Harmful gender norms	5
Santiago Atitlán, community 2	Young adult women	Bad thoughts	Irresponsibility	4
Santiago Atitlán, community 2	Young adult women	Personal characteristics that negatively affect social harmony	Not communicating feelings/ seeking support	4
Santiago Atitlán,	Young adult women	Personal characteristics that negatively affect	Harmful gender norms	5

community 2		social harmony		
Santiago Atitlán, community 2	Young adult women	Poor health promotive care practices	Men's wellbeing	-3
Santiago Atitlán, community 2	Young adult women	Poor health promotive care practices	Poor physical health	3
Santiago Atitlán, community 2	Young adult women	Unwanted pregnancies	Emotional distress	5
Santiago Atitlán, community 2	Young adult women	Lack of formal education	Misuse of technology	4
Santiago Atitlán, community 2	Young adult women	Lack of religious faith	Personal characteristics that negatively affect social harmony	5
Santiago Atitlán, community 2	Young adult women	Lack of religious faith	Low self-esteem	5
Santiago Atitlán, community 2	Young adult women	Disrupted family education	Bad thoughts	5
Santiago Atitlán, community 2	Young adult women	Low self-esteem	Not communicating feelings/ seeking support	4.5
Santiago Atitlán, community 2	Young adult women	Low self-esteem	Bad thoughts	5
Santiago Atitlán, community 2	Young adult women	Low self-esteem	Substance use	5
Santiago Atitlán, community 2	Young adult women	Low self-esteem	Infidelity	5
Santiago Atitlán, community 2	Young adult women	Misuse of technology	Infidelity	5
Santiago Atitlán, community 2	Young adult women	Poor physical health	Men's wellbeing	-5
Santiago Atitlán, community 2	Young adult women	Poor physical health	Basic resource insecurity	4
Santiago Atitlán, community 2	Young adult women	Risk of death	Men's wellbeing	-5

Santiago Atitlán, community 2	Young adult women	Substance use	Men's wellbeing	-5
Santiago Atitlán, community 2	Young adult women	Substance use	Family separation & neglect	5
Santiago Atitlán, community 2	Young adult women	Substance use	Domestic violence	5
Santiago Atitlán, community 2	Young adult women	Substance use	Poor physical health	5
Santiago Atitlán, community 2	Young adult women	Substance use	Poor physical health	5
Santiago Atitlán, community 2	Young adult women	Substance use	Risk of death	5
Santiago Atitlán, community 2	Young adult women	Infidelity	Family separation & neglect	4
Santiago Atitlán, community 2	Young adult women	Infidelity	Low self-esteem	5
Santiago Atitlán, community 2	Young adult women	Infidelity	Substance use	5
Santiago Atitlán, community 2	Older adult men	Emotional distress	Men's wellbeing	-5
Santiago Atitlán, community 2	Older adult men	Emotional distress	Low self-esteem	4
Santiago Atitlán, community 2	Older adult men	Emotional distress	Poor physical health	5
Santiago Atitlán, community 2	Older adult men	Social isolation	Emotional distress	4.5
Santiago Atitlán, community 2	Older adult men	Social isolation	Irresponsibility	3
Santiago Atitlán, community 2	Older adult men	Social isolation	Low self-esteem	4
Santiago	Older adult	Negative social	Emotional distress	4

Atitlán, community 2	men	influences		
Santiago Atitlán, community 2	Older adult men	Basic resource insecurity	Irresponsibility	3
Santiago Atitlán, community 2	Older adult men	Basic resource insecurity	Irresponsibility	-5
Santiago Atitlán, community 2	Older adult men	Basic resource insecurity	Theft	3
Santiago Atitlán, community 2	Older adult men	Basic resource insecurity	Poor health promotive care practices	5
Santiago Atitlán, community 2	Older adult men	Basic resource insecurity	Excessive workload	5
Santiago Atitlán, community 2	Older adult men	Basic resource insecurity	Lack of formal education	5
Santiago Atitlán, community 2	Older adult men	Family separation & neglect	Negative social influences	5
Santiago Atitlán, community 2	Older adult men	Domestic violence	Risk of death	5
Santiago Atitlán, community 2	Older adult men	Irresponsibility	Emotional distress	4
Santiago Atitlán, community 2	Older adult men	Irresponsibility	Basic resource insecurity	4.67
Santiago Atitlán, community 2	Older adult men	Irresponsibility	Personal characteristics that negatively affect social harmony	5
Santiago Atitlán, community 2	Older adult men	Irresponsibility	Unwanted pregnancies	5
Santiago Atitlán, community 2	Older adult men	Irresponsibility	Not respecting customs	5
Santiago Atitlán, community 2	Older adult men	Irresponsibility	Risk of death	5
Santiago Atitlán,	Older adult men	Theft	Risk of death	2

community 2				
Santiago Atitlán, community 2	Older adult men	Personal characteristics that negatively affect social harmony	Emotional distress	4.5
Santiago Atitlán, community 2	Older adult men	Personal characteristics that negatively affect social harmony	Social isolation	5
Santiago Atitlán, community 2	Older adult men	Personal characteristics that negatively affect social harmony	Negative social influences	5
Santiago Atitlán, community 2	Older adult men	Personal characteristics that negatively affect social harmony	Domestic violence	5
Santiago Atitlán, community 2	Older adult men	Personal characteristics that negatively affect social harmony	Irresponsibility	5
Santiago Atitlán, community 2	Older adult men	Personal characteristics that negatively affect social harmony	Lack of religious faith	5
Santiago Atitlán, community 2	Older adult men	Personal characteristics that negatively affect social harmony	Not respecting customs	5
Santiago Atitlán, community 2	Older adult men	Personal characteristics that negatively affect social harmony	Poor physical health	3
Santiago Atitlán, community 2	Older adult men	Personal characteristics that negatively affect social harmony	Risk of death	4
Santiago Atitlán, community 2	Older adult men	Poor health promotive care practices	Poor physical health	4
Santiago Atitlán, community 2	Older adult men	Excessive workload	Poor physical health	4
Santiago Atitlán, community 2	Older adult men	Unwanted pregnancies	Basic resource insecurity	4
Santiago Atitlán, community 2	Older adult men	Unwanted pregnancies	Family separation & neglect	3
Santiago Atitlán, community 2	Older adult men	Lack of formal education	Not respecting customs	-5
Santiago Atitlán, community 2	Older adult men	Lack of formal education	Misuse of technology	-5

Santiago Atitlán, community 2	Older adult men	Lack of formal education	Infidelity	-3
Santiago Atitlán, community 2	Older adult men	Lack of religious faith	Negative social influences	5
Santiago Atitlán, community 2	Older adult men	Lack of religious faith	Not respecting customs	5
Santiago Atitlán, community 2	Older adult men	Lack of religious faith	Risk of death	5
Santiago Atitlán, community 2	Older adult men	Lack of religious faith	Substance use	5
Santiago Atitlán, community 2	Older adult men	Not respecting customs	Emotional distress	4
Santiago Atitlán, community 2	Older adult men	Not respecting customs	Negative social influences	4.5
Santiago Atitlán, community 2	Older adult men	Not respecting customs	Irresponsibility	5
Santiago Atitlán, community 2	Older adult men	Not respecting customs	Personal characteristics that negatively affect social harmony	5
Santiago Atitlán, community 2	Older adult men	Not respecting customs	Excessive workload	5
Santiago Atitlán, community 2	Older adult men	Not respecting customs	Unwanted pregnancies	4
Santiago Atitlán, community 2	Older adult men	Not respecting customs	Lack of religious faith	5
Santiago Atitlán, community 2	Older adult men	Low self-esteem	Emotional distress	5
Santiago Atitlán, community 2	Older adult men	Low self-esteem	Social isolation	4
Santiago Atitlán, community 2	Older adult men	Misuse of technology	Not respecting customs	4
Santiago	Older adult	Misuse of technology	Infidelity	4

Atitlán, community 2	men			
Santiago Atitlán, community 2	Older adult men	Poor physical health	Men's wellbeing	-5
Santiago Atitlán, community 2	Older adult men	Poor physical health	Emotional distress	5
Santiago Atitlán, community 2	Older adult men	Risk of death	Men's wellbeing	-5
Santiago Atitlán, community 2	Older adult men	Substance use	Men's wellbeing	-5
Santiago Atitlán, community 2	Older adult men	Substance use	Family separation & neglect	5
Santiago Atitlán, community 2	Older adult men	Substance use	Domestic violence	5
Santiago Atitlán, community 2	Older adult men	Substance use	Theft	5
Santiago Atitlán, community 2	Older adult men	Substance use	Poor physical health	5
Santiago Atitlán, community 2	Older adult men	Substance use	Risk of death	5
Santiago Atitlán, community 2	Older adult men	Infidelity	Family separation & neglect	5
Santiago Atitlán, community 2	Older adult men	Infidelity	Domestic violence	3
Santiago Atitlán, community 2	Older adult men	Infidelity	Substance use	4
Santiago Atitlán, community 2	Older adult women	Emotional distress	Men's wellbeing	-5
Santiago Atitlán, community 2	Older adult women	Emotional distress	Domestic violence	5
Santiago Atitlán,	Older adult women	Social isolation	Not communicating feelings/ seeking	5

community 2			support	
Santiago Atitlán, community 2	Older adult women	Negative social influences	Lack of affectionate, trusting, supportive family relationships	5
Santiago Atitlán, community 2	Older adult women	Not communicating feelings/ seeking support	Emotional distress	4
Santiago Atitlán, community 2	Older adult women	Not communicating feelings/ seeking support	Domestic violence	5
Santiago Atitlán, community 2	Older adult women	Not communicating feelings/ seeking support	Substance use	4.75
Santiago Atitlán, community 2	Older adult women	Basic resource insecurity	Unemployment	3
Santiago Atitlán, community 2	Older adult women	Basic resource insecurity	Theft	5
Santiago Atitlán, community 2	Older adult women	Unemployment	Basic resource insecurity	3
Santiago Atitlán, community 2	Older adult women	Unemployment	Bad thoughts	5
Santiago Atitlán, community 2	Older adult women	Unemployment	Misuse of technology	3
Santiago Atitlán, community 2	Older adult women	Family separation & neglect	Negative social influences	5
Santiago Atitlán, community 2	Older adult women	Domestic violence	Men's wellbeing	-5
Santiago Atitlán, community 2	Older adult women	Domestic violence	Irresponsibility	3
Santiago Atitlán, community 2	Older adult women	Domestic violence	Personal characteristics that negatively affect social harmony	4
Santiago Atitlán, community 2	Older adult women	Domestic violence	Risk of death	5
Santiago Atitlán, community 2	Older adult women	Lack of affectionate, trusting, supportive family relationships	Substance use	5

Santiago Atitlán, community 2	Older adult women	Irresponsibility	Basic resource insecurity	5
Santiago Atitlán, community 2	Older adult women	Irresponsibility	Unemployment	4
Santiago Atitlán, community 2	Older adult women	Irresponsibility	Family separation & neglect	3
Santiago Atitlán, community 2	Older adult women	Irresponsibility	Lack of affectionate, trusting, supportive family relationships	3
Santiago Atitlán, community 2	Older adult women	Irresponsibility	Theft	3.5
Santiago Atitlán, community 2	Older adult women	Irresponsibility	Bad thoughts	4
Santiago Atitlán, community 2	Older adult women	Irresponsibility	Personal characteristics that negatively affect social harmony	5
Santiago Atitlán, community 2	Older adult women	Theft	Unemployment	3
Santiago Atitlán, community 2	Older adult women	Theft	Domestic violence	4
Santiago Atitlán, community 2	Older adult women	Theft	Risk of death	5
Santiago Atitlán, community 2	Older adult women	Bad thoughts	Men's wellbeing	-4
Santiago Atitlán, community 2	Older adult women	Bad thoughts	Basic resource insecurity	4
Santiago Atitlán, community 2	Older adult women	Bad thoughts	Lack of affectionate, trusting, supportive family relationships	5
Santiago Atitlán, community 2	Older adult women	Personal characteristics that negatively affect social harmony	Emotional distress	4
Santiago Atitlán, community 2	Older adult women	Personal characteristics that negatively affect social harmony	Social isolation	4.5
Santiago	Older adult	Personal characteristics	Negative social	5

Atitlán, community 2	women	that negatively affect social harmony	influences	
Santiago Atitlán, community 2	Older adult women	Personal characteristics that negatively affect social harmony	Lack of affectionate, trusting, supportive family relationships	5
Santiago Atitlán, community 2	Older adult women	Personal characteristics that negatively affect social harmony	Irresponsibility	5
Santiago Atitlán, community 2	Older adult women	Personal characteristics that negatively affect social harmony	Bad thoughts	5
Santiago Atitlán, community 2	Older adult women	Personal characteristics that negatively affect social harmony	Lack of religious faith	5
Santiago Atitlán, community 2	Older adult women	Personal characteristics that negatively affect social harmony	Misuse of technology	3
Santiago Atitlán, community 2	Older adult women	Personal characteristics that negatively affect social harmony	Infidelity	5
Santiago Atitlán, community 2	Older adult women	Unwanted pregnancies	Lack of affectionate, trusting, supportive family relationships	4
Santiago Atitlán, community 2	Older adult women	Lack of religious faith	Negative social influences	5
Santiago Atitlán, community 2	Older adult women	Lack of religious faith	Basic resource insecurity	4
Santiago Atitlán, community 2	Older adult women	Lack of religious faith	Personal characteristics that negatively affect social harmony	5
Santiago Atitlán, community 2	Older adult women	Lack of religious faith	Risk of death	5
Santiago Atitlán, community 2	Older adult women	Lack of religious faith	Substance use	5
Santiago Atitlán, community 2	Older adult women	Lack of religious faith	Infidelity	5
Santiago Atitlán, community 2	Older adult women	Misuse of technology	Irresponsibility	5
Santiago Atitlán,	Older adult women	Misuse of technology	Bad thoughts	4

community 2				
Santiago Atitlán, community 2	Older adult women	Misuse of technology	Infidelity	3
Santiago Atitlán, community 2	Older adult women	Poor physical health	Men's wellbeing	-3
Santiago Atitlán, community 2	Older adult women	Poor physical health	Risk of death	3
Santiago Atitlán, community 2	Older adult women	Risk of death	Men's wellbeing	-5
Santiago Atitlán, community 2	Older adult women	Substance use	Men's wellbeing	-5
Santiago Atitlán, community 2	Older adult women	Substance use	Domestic violence	5
Santiago Atitlán, community 2	Older adult women	Substance use	Irresponsibility	3.5
Santiago Atitlán, community 2	Older adult women	Substance use	Theft	5
Santiago Atitlán, community 2	Older adult women	Substance use	Poor physical health	3
Santiago Atitlán, community 2	Older adult women	Substance use	Risk of death	4
Santiago Atitlán, community 2	Older adult women	Infidelity	Family separation & neglect	5
Santiago Atitlán, community 2	Older adult women	Infidelity	Lack of affectionate, trusting, supportive family relationships	5
Santiago Atitlán, community 2	Older adult women	Infidelity	Unwanted pregnancies	5
Santiago Atitlán, community 2	Older adult women	Infidelity	Poor physical health	4
Santiago Atitlán, community 2	Older adult women	Infidelity	Substance use	5

Cuilco, community 1	Adult men	Emotional distress	Substance use	5
Cuilco, community 1	Adult men	Emotional distress	Not sleeping well	5
Cuilco, community 1	Adult men	Emotional distress	Men's wellbeing	-5
Cuilco, community 1	Adult men	Not communicating feelings/ seeking support	Substance use	3
Cuilco, community 1	Adult men	Not communicating feelings/ seeking support	Personal characteristics that negatively affect social harmony	5
Cuilco, community 1	Adult men	Not communicating feelings/ seeking support	Emotional distress	5
Cuilco, community 1	Adult men	Not communicating feelings/ seeking support	Domestic violence	5
Cuilco, community 1	Adult men	Basic resource insecurity	Lack of affectionate, trusting, supportive family relationships	5
Cuilco, community 1	Adult men	Basic resource insecurity	Poor physical health	5
Cuilco, community 1	Adult men	Basic resource insecurity	Excessive workload	5
Cuilco, community 1	Adult men	Basic resource insecurity	Poor health promotive care practices	5
Cuilco, community 1	Adult men	Unemployment	Basic resource insecurity	5
Cuilco, community 1	Adult men	Unemployment	Not sleeping well	5
Cuilco, community 1	Adult men	Migration	Lack of affectionate, trusting, supportive family relationships	5
Cuilco, community 1	Adult men	Migration	Family separation & neglect	5
Cuilco, community 1	Adult men	Family separation & neglect	Emotional distress	5
Cuilco, community 1	Adult men	Family separation & neglect	Substance use	4
Cuilco, community 1	Adult men	Domestic violence	Emotional distress	5
Cuilco, community 1	Adult men	Domestic violence	Family separation & neglect	5
Cuilco, community 1	Adult men	Lack of affectionate, trusting, supportive	Emotional distress	5

		family relationships		
Cuilco, community 1	Adult men	Lack of affectionate, trusting, supportive family relationships	Not sleeping well	5
Cuilco, community 1	Adult men	Lack of affectionate, trusting, supportive family relationships	Infidelity	3
Cuilco, community 1	Adult men	Lack of affectionate, trusting, supportive family relationships	Low self-esteem	4
Cuilco, community 1	Adult men	Lack of affectionate, trusting, supportive family relationships	Men's wellbeing	-5
Cuilco, community 1	Adult men	Lack of affectionate, trusting, supportive family relationships	Domestic violence	5
Cuilco, community 1	Adult men	Harmful gender norms	Domestic violence	5
Cuilco, community 1	Adult men	Harmful gender norms	Not communicating feelings/ seeking support	5
Cuilco, community 1	Adult men	Harmful gender norms	Personal characteristics that negatively affect social harmony	4
Cuilco, community 1	Adult men	Irresponsibility	Basic resource insecurity	3
Cuilco, community 1	Adult men	Personal characteristics that negatively affect social harmony	Lack of affectionate, trusting, supportive family relationships	5
Cuilco, community 1	Adult men	Personal characteristics that negatively affect social harmony	Domestic violence	5
Cuilco, community 1	Adult men	Poor health promotive care practices	Men's wellbeing	-5
Cuilco, community 1	Adult men	Poor health promotive care practices	Sports/recreation	-3
Cuilco, community 1	Adult men	Poor health promotive care practices	Poor physical health	4.83
Cuilco, community 1	Adult men	Excessive workload	Not sleeping well	3
Cuilco, community 1	Adult men	Excessive workload	Poor physical health	3
Cuilco, community 1	Adult men	Excessive workload	Sports/recreation	-3
Cuilco, community 1	Adult men	Sports/recreation	Emotional distress	-3

Cuilco, community 1	Adult men	Sports/recreation	Men's wellbeing	3
Cuilco, community 1	Adult men	Sports/recreation	Poor physical health	-3
Cuilco, community 1	Adult men	Not sleeping well	Emotional distress	4
Cuilco, community 1	Adult men	Unwanted pregnancies	Basic resource insecurity	3
Cuilco, community 1	Adult men	Unwanted pregnancies	Emotional distress	3
Cuilco, community 1	Adult men	Low self-esteem	Lack of affectionate, trusting, supportive family relationships	5
Cuilco, community 1	Adult men	Low self-esteem	Suicidality	3
Cuilco, community 1	Adult men	Poor physical health	Sports/recreation	-2.5
Cuilco, community 1	Adult men	Poor physical health	Lack of affectionate, trusting, supportive family relationships	5
Cuilco, community 1	Adult men	Poor physical health	Family separation & neglect	5
Cuilco, community 1	Adult men	Poor physical health	Emotional distress	5
Cuilco, community 1	Adult men	Poor physical health	Men's wellbeing	-5
Cuilco, community 1	Adult men	Substance use	Men's wellbeing	-5
Cuilco, community 1	Adult men	Substance use	Low self-esteem	3
Cuilco, community 1	Adult men	Substance use	Family separation & neglect	3.5
Cuilco, community 1	Adult men	Substance use	Basic resource insecurity	5
Cuilco, community 1	Adult men	Substance use	Domestic violence	5
Cuilco, community 1	Adult men	Suicidality	Men's wellbeing	-5
Cuilco, community 1	Adult men	Infidelity	Family separation & neglect	4
Cuilco, community 1	Adult men	Infidelity	Migration	5
Cuilco, community 1	Adult men	Infidelity	Poor physical health	5
Cuilco, community 1	Adult men	Infidelity	Unwanted pregnancies	5

Cuilco, community 1	Adult women	Emotional distress	Men's wellbeing	-5
Cuilco, community 1	Adult women	Emotional distress	Poor physical health	3
Cuilco, community 1	Adult women	Emotional distress	Substance use	4
Cuilco, community 1	Adult women	Negative social influences	Lack of affectionate, trusting, supportive family relationships	3
Cuilco, community 1	Adult women	Negative social influences	Substance use	3
Cuilco, community 1	Adult women	Basic resource insecurity	Emotional distress	5
Cuilco, community 1	Adult women	Basic resource insecurity	Excessive workload	5
Cuilco, community 1	Adult women	Basic resource insecurity	Poor physical health	4
Cuilco, community 1	Adult women	Unemployment	Emotional distress	4.5
Cuilco, community 1	Adult women	Unemployment	Basic resource insecurity	4.5
Cuilco, community 1	Adult women	Unemployment	Lack of affectionate, trusting, supportive family relationships	3
Cuilco, community 1	Adult women	Unemployment	Lack of formal education	4
Cuilco, community 1	Adult women	Unemployment	Poor physical health	4
Cuilco, community 1	Adult women	Domestic violence	Lack of affectionate, trusting, supportive family relationships	5
Cuilco, community 1	Adult women	Domestic violence	Unwanted pregnancies	4
Cuilco, community 1	Adult women	Domestic violence	Poor physical health	4
Cuilco, community 1	Adult women	Lack of affectionate, trusting, supportive family relationships	Emotional distress	3
Cuilco, community 1	Adult women	Lack of affectionate, trusting, supportive family relationships	Negative social influences	4
Cuilco, community 1	Adult women	Lack of affectionate, trusting, supportive family relationships	Unemployment	5
Cuilco, community 1	Adult women	Lack of affectionate, trusting, supportive	Personal characteristics that negatively affect	3

		family relationships	social harmony	
Cuilco, community 1	Adult women	Lack of affectionate, trusting, supportive family relationships	Lack of formal education	4
Cuilco, community 1	Adult women	Lack of affectionate, trusting, supportive family relationships	Poor physical health	3.33
Cuilco, community 1	Adult women	Lack of affectionate, trusting, supportive family relationships	Suicidality	3
Cuilco, community 1	Adult women	Lack of affectionate, trusting, supportive family relationships	Infidelity	3.5
Cuilco, community 1	Adult women	Personal characteristics that negatively affect social harmony	Domestic violence	3
Cuilco, community 1	Adult women	Personal characteristics that negatively affect social harmony	Lack of affectionate, trusting, supportive family relationships	4
Cuilco, community 1	Adult women	Excessive workload	Emotional distress	3
Cuilco, community 1	Adult women	Excessive workload	Lack of affectionate, trusting, supportive family relationships	3
Cuilco, community 1	Adult women	Excessive workload	Poor physical health	5
Cuilco, community 1	Adult women	Unwanted pregnancies	Emotional distress	5
Cuilco, community 1	Adult women	Unwanted pregnancies	Basic resource insecurity	5
Cuilco, community 1	Adult women	Unwanted pregnancies	Domestic violence	3
Cuilco, community 1	Adult women	Lack of formal education	Unemployment	4.5
Cuilco, community 1	Adult women	Lack of formal education	Lack of affectionate, trusting, supportive family relationships	3
Cuilco, community 1	Adult women	Poor physical health	Men's wellbeing	-4.83
Cuilco, community 1	Adult women	Poor physical health	Emotional distress	5
Cuilco, community 1	Adult women	Poor physical health	Risk of death	4
Cuilco, community 1	Adult women	Risk of death	Emotional distress	5

Cuilco, community 1	Adult women	Risk of death	Substance use	5
Cuilco, community 1	Adult women	Substance use	Basic resource insecurity	5
Cuilco, community 1	Adult women	Substance use	Lack of affectionate, trusting, supportive family relationships	5
Cuilco, community 1	Adult women	Substance use	Personal characteristics that negatively affect social harmony	2
Cuilco, community 1	Adult women	Substance use	Poor physical health	4
Cuilco, community 1	Adult women	Substance use	Suicidality	3
Cuilco, community 1	Adult women	Suicidality	Men's wellbeing	-5
Cuilco, community 1	Adult women	Infidelity	Basic resource insecurity	5
Cuilco, community 1	Adult women	Infidelity	Lack of affectionate, trusting, supportive family relationships	4
Cuilco, community 1	Adult women	Infidelity	Unwanted pregnancies	3
Cuilco, community 1	Adult women	Infidelity	Poor physical health	5
Cuilco, community 1	*Terapeutas tradicionales*	Emotional distress	Men's wellbeing	-5
Cuilco, community 1	*Terapeutas tradicionales*	Emotional distress	Domestic violence	5
Cuilco, community 1	*Terapeutas tradicionales*	Emotional distress	Lack of affectionate, trusting, supportive family relationships	5
Cuilco, community 1	*Terapeutas tradicionales*	Emotional distress	Poor physical health	5
Cuilco, community 1	*Terapeutas tradicionales*	Negative social influences	Emotional distress	5
Cuilco, community 1	*Terapeutas tradicionales*	Negative social influences	Domestic violence	4
Cuilco, community 1	*Terapeutas tradicionales*	Negative social influences	Personal characteristics that negatively affect social harmony	4
Cuilco, community 1	*Terapeutas tradicionales*	Negative social influences	Substance use	3
Cuilco, community 1	*Terapeutas tradicionales*	Basic resource insecurity	Men's wellbeing	-3
Cuilco,	*Terapeutas*	Basic resource	Emotional distress	5

community 1	*tradicionales*	insecurity		
Cuilco, community 1	*Terapeutas tradicionales*	Basic resource insecurity	Migration	5
Cuilco, community 1	*Terapeutas tradicionales*	Basic resource insecurity	Lack of affectionate, trusting, supportive family relationships	5
Cuilco, community 1	*Terapeutas tradicionales*	Basic resource insecurity	Poor health promotive care practices	5
Cuilco, community 1	*Terapeutas tradicionales*	Basic resource insecurity	Lack of formal education	5
Cuilco, community 1	*Terapeutas tradicionales*	Basic resource insecurity	Poor physical health	5
Cuilco, community 1	*Terapeutas tradicionales*	Unemployment	Basic resource insecurity	5
Cuilco, community 1	*Terapeutas tradicionales*	Unemployment	Migration	5
Cuilco, community 1	*Terapeutas tradicionales*	Migration	Basic resource insecurity	4
Cuilco, community 1	*Terapeutas tradicionales*	Migration	Family separation & neglect	5
Cuilco, community 1	*Terapeutas tradicionales*	Migration	Lack of affectionate, trusting, supportive family relationships	4
Cuilco, community 1	*Terapeutas tradicionales*	Family separation & neglect	Emotional distress	3
Cuilco, community 1	*Terapeutas tradicionales*	Domestic violence	Men's wellbeing	-5
Cuilco, community 1	*Terapeutas tradicionales*	Domestic violence	Family separation & neglect	5
Cuilco, community 1	*Terapeutas tradicionales*	Domestic violence	Lack of affectionate, trusting, supportive family relationships	4
Cuilco, community 1	*Terapeutas tradicionales*	Domestic violence	Poor physical health	5
Cuilco, community 1	*Terapeutas tradicionales*	Domestic violence	Substance use	3.5
Cuilco, community 1	*Terapeutas tradicionales*	Lack of affectionate, trusting, supportive family relationships	Men's wellbeing	-5
Cuilco, community 1	*Terapeutas tradicionales*	Lack of affectionate, trusting, supportive family relationships	Migration	3
Cuilco, community 1	*Terapeutas tradicionales*	Lack of affectionate, trusting, supportive family relationships	Family separation & neglect	4

Cuilco, community 1	*Terapeutas tradicionales*	Lack of affectionate, trusting, supportive family relationships	Domestic violence	4.25
Cuilco, community 1	*Terapeutas tradicionales*	Lack of affectionate, trusting, supportive family relationships	Bad thoughts	5
Cuilco, community 1	*Terapeutas tradicionales*	Lack of affectionate, trusting, supportive family relationships	Unwanted pregnancies	5
Cuilco, community 1	*Terapeutas tradicionales*	Lack of affectionate, trusting, supportive family relationships	Substance use	5
Cuilco, community 1	*Terapeutas tradicionales*	Lack of affectionate, trusting, supportive family relationships	Infidelity	5
Cuilco, community 1	*Terapeutas tradicionales*	Harmful gender norms	Lack of affectionate, trusting, supportive family relationships	4
Cuilco, community 1	*Terapeutas tradicionales*	Harmful gender norms	Unwanted pregnancies	5
Cuilco, community 1	*Terapeutas tradicionales*	Harmful gender norms	Infidelity	5
Cuilco, community 1	*Terapeutas tradicionales*	Irresponsibility	Emotional distress	5
Cuilco, community 1	*Terapeutas tradicionales*	Irresponsibility	Negative social influences	5
Cuilco, community 1	*Terapeutas tradicionales*	Irresponsibility	Basic resource insecurity	4
Cuilco, community 1	*Terapeutas tradicionales*	Irresponsibility	Bad thoughts	5
Cuilco, community 1	*Terapeutas tradicionales*	Irresponsibility	Personal characteristics that negatively affect social harmony	5
Cuilco, community 1	*Terapeutas tradicionales*	Irresponsibility	Poor health promotive care practices	3.75
Cuilco, community 1	*Terapeutas tradicionales*	Irresponsibility	Taking care of the environment	-5
Cuilco, community 1	*Terapeutas tradicionales*	Irresponsibility	Substance use	5
Cuilco, community 1	*Terapeutas tradicionales*	Irresponsibility	Infidelity	5
Cuilco, community 1	*Terapeutas tradicionales*	Bad thoughts	Emotional distress	5
Cuilco, community 1	*Terapeutas tradicionales*	Bad thoughts	Lack of affectionate, trusting, supportive family relationships	3

Cuilco, community 1	*Terapeutas tradicionales*	Bad thoughts	Infidelity	3
Cuilco, community 1	*Terapeutas tradicionales*	Personal characteristics that negatively affect social harmony	Negative social influences	4
Cuilco, community 1	*Terapeutas tradicionales*	Personal characteristics that negatively affect social harmony	Domestic violence	5
Cuilco, community 1	*Terapeutas tradicionales*	Personal characteristics that negatively affect social harmony	Lack of affectionate, trusting, supportive family relationships	5
Cuilco, community 1	*Terapeutas tradicionales*	Personal characteristics that negatively affect social harmony	Irresponsibility	4
Cuilco, community 1	*Terapeutas tradicionales*	Personal characteristics that negatively affect social harmony	Substance use	3
Cuilco, community 1	*Terapeutas tradicionales*	Poor health promotive care practices	Poor physical health	5
Cuilco, community 1	*Terapeutas tradicionales*	Excessive workload	Poor physical health	4
Cuilco, community 1	*Terapeutas tradicionales*	Taking care of the environment	Poor physical health	-5
Cuilco, community 1	*Terapeutas tradicionales*	Unwanted pregnancies	Emotional distress	5
Cuilco, community 1	*Terapeutas tradicionales*	Unwanted pregnancies	Basic resource insecurity	4.67
Cuilco, community 1	*Terapeutas tradicionales*	Unwanted pregnancies	Family separation & neglect	5
Cuilco, community 1	*Terapeutas tradicionales*	Lack of formal education	Basic resource insecurity	3
Cuilco, community 1	*Terapeutas tradicionales*	Poor physical health	Men's wellbeing	-5
Cuilco, community 1	*Terapeutas tradicionales*	Substance use	Men's wellbeing	-5
Cuilco, community 1	*Terapeutas tradicionales*	Substance use	Irresponsibility	5
Cuilco, community 1	*Terapeutas tradicionales*	Substance use	Poor physical health	5
Cuilco, community 1	*Terapeutas tradicionales*	Infidelity	Family separation & neglect	4
Cuilco, community 1	*Terapeutas tradicionales*	Infidelity	Lack of affectionate, trusting, supportive family relationships	4.5
Cuilco, community 1	*Terapeutas tradicionales*	Infidelity	Unwanted pregnancies	5

Cuilco, community 1	*Terapeutas tradicionales*	Infidelity	Poor physical health	5
Cuilco, community 2	Adult men	Emotional distress	Men's wellbeing	-5
Cuilco, community 2	Adult men	Emotional distress	Lack of affectionate, trusting, supportive family relationships	3
Cuilco, community 2	Adult men	Emotional distress	Irresponsibility	4
Cuilco, community 2	Adult men	Emotional distress	Poor health promotive care practices	2
Cuilco, community 2	Adult men	Emotional distress	Poor physical health	4
Cuilco, community 2	Adult men	Emotional distress	Infidelity	5
Cuilco, community 2	Adult men	Negative social influences	Substance use	3
Cuilco, community 2	Adult men	Basic resource insecurity	Emotional distress	3.83
Cuilco, community 2	Adult men	Basic resource insecurity	Poor health promotive care practices	4.5
Cuilco, community 2	Adult men	Basic resource insecurity	Excessive workload	5
Cuilco, community 2	Adult men	Unemployment	Basic resource insecurity	5
Cuilco, community 2	Adult men	Domestic violence	Unwanted pregnancies	3
Cuilco, community 2	Adult men	Domestic violence	Poor physical health	3
Cuilco, community 2	Adult men	Lack of affectionate, trusting, supportive family relationships	Domestic violence	3.5
Cuilco, community 2	Adult men	Lack of affectionate, trusting, supportive family relationships	Personal characteristics that negatively affect social harmony	3
Cuilco, community 2	Adult men	Lack of affectionate, trusting, supportive family relationships	Low self-esteem	3
Cuilco, community 2	Adult men	Harmful gender norms	Domestic violence	5
Cuilco, community 2	Adult men	Harmful gender norms	Lack of affectionate, trusting, supportive family relationships	5
Cuilco, community 2	Adult men	Harmful gender norms	Infidelity	3
Cuilco,	Adult men	Irresponsibility	Basic resource	4

community 2			insecurity	
Cuilco, community 2	Adult men	Irresponsibility	Domestic violence	5
Cuilco, community 2	Adult men	Irresponsibility	Lack of affectionate, trusting, supportive family relationships	3
Cuilco, community 2	Adult men	Irresponsibility	Poor health promotive care practices	3
Cuilco, community 2	Adult men	Irresponsibility	Taking care of the environment	-5
Cuilco, community 2	Adult men	Irresponsibility	Poor physical health	5
Cuilco, community 2	Adult men	Bad thoughts	Lack of affectionate, trusting, supportive family relationships	5
Cuilco, community 2	Adult men	Bad thoughts	Substance use	5
Cuilco, community 2	Adult men	Personal characteristics that negatively affect social harmony	Lack of affectionate, trusting, supportive family relationships	5
Cuilco, community 2	Adult men	Personal characteristics that negatively affect social harmony	Domestic violence	4
Cuilco, community 2	Adult men	Poor health promotive care practices	Poor physical health	5
Cuilco, community 2	Adult men	Excessive workload	Men's wellbeing	-3
Cuilco, community 2	Adult men	Excessive workload	Not sleeping well	4
Cuilco, community 2	Adult men	Excessive workload	Poor physical health	2.5
Cuilco, community 2	Adult men	Not sleeping well	Emotional distress	3
Cuilco, community 2	Adult men	Not sleeping well	Poor physical health	3
Cuilco, community 2	Adult men	Taking care of the environment	Poor physical health	-3
Cuilco, community 2	Adult men	Unwanted pregnancies	Bad thoughts	4
Cuilco, community 2	Adult men	Lack of formal education	Emotional distress	5
Cuilco, community 2	Adult men	Lack of formal education	Basic resource insecurity	5
Cuilco, community 2	Adult men	Lack of formal education	Unemployment	4

Cuilco, community 2	Adult men	Lack of formal education	Harmful gender norms	4
Cuilco, community 2	Adult men	Lack of formal education	Irresponsibility	3.4
Cuilco, community 2	Adult men	Lack of formal education	Poor health promotive care practices	3
Cuilco, community 2	Adult men	Lack of formal education	Substance use	5
Cuilco, community 2	Adult men	Low self-esteem	Bad thoughts	5
Cuilco, community 2	Adult men	Poor physical health	Men's wellbeing	-4.33
Cuilco, community 2	Adult men	Poor physical health	Lack of affectionate, trusting, supportive family relationships	4
Cuilco, community 2	Adult men	Substance use	Emotional distress	5
Cuilco, community 2	Adult men	Substance use	Basic resource insecurity	4
Cuilco, community 2	Adult men	Substance use	Poor physical health	3
Cuilco, community 2	Adult men	Infidelity	Domestic violence	3
Cuilco, community 2	Adult men	Infidelity	Unwanted pregnancies	5
Cuilco, community 2	Adult men	Infidelity	Poor physical health	5
Cuilco, community 2	Adult women	Emotional distress	Men's wellbeing	-4.67
Cuilco, community 2	Adult women	Emotional distress	Domestic violence	5
Cuilco, community 2	Adult women	Emotional distress	Substance use	3
Cuilco, community 2	Adult women	Emotional distress	Suicidality	5
Cuilco, community 2	Adult women	Negative social influences	Substance use	3.5
Cuilco, community 2	Adult women	Basic resource insecurity	Emotional distress	4.67
Cuilco, community 2	Adult women	Basic resource insecurity	Poor health promotive care practices	5
Cuilco, community 2	Adult women	Basic resource insecurity	Poor physical health	4.33
Cuilco, community 2	Adult women	Basic resource insecurity	Substance use	-3

Cuilco, community 2	Adult women	Unemployment	Emotional distress	3
Cuilco, community 2	Adult women	Unemployment	Basic resource insecurity	4.83
Cuilco, community 2	Adult women	Unemployment	Migration	5
Cuilco, community 2	Adult women	Migration	Family separation & neglect	5
Cuilco, community 2	Adult women	Migration	Lack of affectionate, trusting, supportive family relationships	2
Cuilco, community 2	Adult women	Family separation & neglect	Emotional distress	3
Cuilco, community 2	Adult women	Family separation & neglect	Self-care	5
Cuilco, community 2	Adult women	Domestic violence	Emotional distress	5
Cuilco, community 2	Adult women	Domestic violence	Lack of affectionate, trusting, supportive family relationships	5
Cuilco, community 2	Adult women	Domestic violence	Prison	4
Cuilco, community 2	Adult women	Lack of affectionate, trusting, supportive family relationships	Emotional distress	3.86
Cuilco, community 2	Adult women	Lack of affectionate, trusting, supportive family relationships	Family separation & neglect	5
Cuilco, community 2	Adult women	Lack of affectionate, trusting, supportive family relationships	Domestic violence	5
Cuilco, community 2	Adult women	Lack of affectionate, trusting, supportive family relationships	Bad thoughts	4
Cuilco, community 2	Adult women	Lack of affectionate, trusting, supportive family relationships	Self-care	3
Cuilco, community 2	Adult women	Lack of affectionate, trusting, supportive family relationships	Poor physical health	3
Cuilco, community 2	Adult women	Lack of affectionate, trusting, supportive family relationships	Substance use	3.5
Cuilco, community 2	Adult women	Lack of affectionate, trusting, supportive family relationships	Suicidality	5

Cuilco, community 2	Adult women	Lack of affectionate, trusting, supportive family relationships	Infidelity	1.5
Cuilco, community 2	Adult women	Harmful gender norms	Emotional distress	5
Cuilco, community 2	Adult women	Harmful gender norms	Lack of affectionate, trusting, supportive family relationships	3
Cuilco, community 2	Adult women	Harmful gender norms	Unwanted pregnancies	5
Cuilco, community 2	Adult women	Harmful gender norms	Substance use	5
Cuilco, community 2	Adult women	Irresponsibility	Basic resource insecurity	4.5
Cuilco, community 2	Adult women	Theft	Domestic violence	5
Cuilco, community 2	Adult women	Theft	Prison	5
Cuilco, community 2	Adult women	Prison	Emotional distress	4
Cuilco, community 2	Adult women	Prison	Poor physical health	5
Cuilco, community 2	Adult women	Bad thoughts	Lack of affectionate, trusting, supportive family relationships	5
Cuilco, community 2	Adult women	Bad thoughts	Substance use	3
Cuilco, community 2	Adult women	Poor health promotive care practices	Men's wellbeing	-5
Cuilco, community 2	Adult women	Poor health promotive care practices	Emotional distress	4
Cuilco, community 2	Adult women	Poor health promotive care practices	Poor physical health	5
Cuilco, community 2	Adult women	Self-care	Emotional distress	5
Cuilco, community 2	Adult women	Excessive workload	Poor physical health	5
Cuilco, community 2	Adult women	Sports/recreation	Emotional distress	-5
Cuilco, community 2	Adult women	Sports/recreation	Domestic violence	-3
Cuilco, community 2	Adult women	Sports/recreation	Lack of affectionate, trusting, supportive family relationships	-3
Cuilco, community 2	Adult women	Sports/recreation	Substance use	-4

Cuilco, community 2	Adult women	Unwanted pregnancies	Emotional distress	5
Cuilco, community 2	Adult women	Unwanted pregnancies	Migration	5
Cuilco, community 2	Adult women	Unwanted pregnancies	Excessive workload	5
Cuilco, community 2	Adult women	Poor physical health	Men's wellbeing	-5
Cuilco, community 2	Adult women	Poor physical health	Emotional distress	5
Cuilco, community 2	Adult women	Substance use	Emotional distress	3
Cuilco, community 2	Adult women	Substance use	Domestic violence	5
Cuilco, community 2	Adult women	Substance use	Irresponsibility	5
Cuilco, community 2	Adult women	Substance use	Theft	5
Cuilco, community 2	Adult women	Substance use	Self-care	3.5
Cuilco, community 2	Adult women	Substance use	Poor physical health	5
Cuilco, community 2	Adult women	Suicidality	Men's wellbeing	-5
Cuilco, community 2	Adult women	Infidelity	Family separation & neglect	4
Cuilco, community 2	Adult women	Infidelity	Lack of affectionate, trusting, supportive family relationships	4.75
Cuilco, community 2	Adult women	Infidelity	Poor physical health	5
Cuilco, community 2	Adult women	Infidelity	Substance use	5
Cuilco, community 2	Adult women	Infidelity	Suicidality	5
Cuilco, community 2	*Terapeutas tradicionales*	Emotional distress	Men's wellbeing	-4.75
Cuilco, community 2	*Terapeutas tradicionales*	Negative social influences	Theft	3
Cuilco, community 2	*Terapeutas tradicionales*	Negative social influences	Misuse of technology	3
Cuilco, community 2	*Terapeutas tradicionales*	Negative social influences	Substance use	4.33
Cuilco, community 2	*Terapeutas tradicionales*	Basic resource insecurity	Emotional distress	4.67

Cuilco, community 2	*Terapeutas tradicionales*	Basic resource insecurity	Poor health promotive care practices	5
Cuilco, community 2	*Terapeutas tradicionales*	Basic resource insecurity	Self-care	3.5
Cuilco, community 2	*Terapeutas tradicionales*	Unemployment	Emotional distress	5
Cuilco, community 2	*Terapeutas tradicionales*	Unemployment	Basic resource insecurity	5
Cuilco, community 2	*Terapeutas tradicionales*	Unemployment	Poor health promotive care practices	5
Cuilco, community 2	*Terapeutas tradicionales*	Unemployment	Self-care	5
Cuilco, community 2	*Terapeutas tradicionales*	Unemployment	Sports/recreation	-4
Cuilco, community 2	*Terapeutas tradicionales*	Domestic violence	Men's wellbeing	-5
Cuilco, community 2	*Terapeutas tradicionales*	Domestic violence	Emotional distress	4.67
Cuilco, community 2	*Terapeutas tradicionales*	Domestic violence	Lack of affectionate, trusting, supportive family relationships	5
Cuilco, community 2	*Terapeutas tradicionales*	Lack of affectionate, trusting, supportive family relationships	Men's wellbeing	-4
Cuilco, community 2	*Terapeutas tradicionales*	Lack of affectionate, trusting, supportive family relationships	Emotional distress	4
Cuilco, community 2	*Terapeutas tradicionales*	Lack of affectionate, trusting, supportive family relationships	Domestic violence	4
Cuilco, community 2	*Terapeutas tradicionales*	Lack of affectionate, trusting, supportive family relationships	Irresponsibility	3
Cuilco, community 2	*Terapeutas tradicionales*	Lack of affectionate, trusting, supportive family relationships	Substance use	4.33
Cuilco, community 2	*Terapeutas tradicionales*	Harmful gender norms	Domestic violence	5
Cuilco, community 2	*Terapeutas tradicionales*	Harmful gender norms	Lack of affectionate, trusting, supportive family relationships	4
Cuilco, community 2	*Terapeutas tradicionales*	Harmful gender norms	Irresponsibility	3
Cuilco, community 2	*Terapeutas tradicionales*	Harmful gender norms	Infidelity	5
Cuilco,	*Terapeutas*	Irresponsibility	Basic resource	5

community 2	*tradicionales*		insecurity	
Cuilco, community 2	*Terapeutas tradicionales*	Theft	Domestic violence	5
Cuilco, community 2	*Terapeutas tradicionales*	Theft	Prison	3.5
Cuilco, community 2	*Terapeutas tradicionales*	Prison	Emotional distress	3
Cuilco, community 2	*Terapeutas tradicionales*	Prison	Domestic violence	4
Cuilco, community 2	*Terapeutas tradicionales*	Prison	Lack of affectionate, trusting, supportive family relationships	5
Cuilco, community 2	*Terapeutas tradicionales*	Personal characteristics that negatively affect social harmony	Men's wellbeing	-5
Cuilco, community 2	*Terapeutas tradicionales*	Personal characteristics that negatively affect social harmony	Emotional distress	4
Cuilco, community 2	*Terapeutas tradicionales*	Personal characteristics that negatively affect social harmony	Lack of affectionate, trusting, supportive family relationships	4.5
Cuilco, community 2	*Terapeutas tradicionales*	Personal characteristics that negatively affect social harmony	Harmful gender norms	5
Cuilco, community 2	*Terapeutas tradicionales*	Personal characteristics that negatively affect social harmony	Substance use	5
Cuilco, community 2	*Terapeutas tradicionales*	Poor health promotive care practices	Men's wellbeing	-5
Cuilco, community 2	*Terapeutas tradicionales*	Poor health promotive care practices	Emotional distress	4
Cuilco, community 2	*Terapeutas tradicionales*	Self-care	Personal characteristics that negatively affect social harmony	4.5
Cuilco, community 2	*Terapeutas tradicionales*	Self-care	Sports/recreation	-4
Cuilco, community 2	*Terapeutas tradicionales*	Self-care	Lack of religious faith	5
Cuilco, community 2	*Terapeutas tradicionales*	Sports/recreation	Personal characteristics that negatively affect social harmony	-4
Cuilco, community 2	*Terapeutas tradicionales*	Lack of religious faith	Personal characteristics that negatively affect social harmony	5
Cuilco, community 2	*Terapeutas tradicionales*	Misuse of technology	Emotional distress	5

Cuilco, community 2	*Terapeutas tradicionales*	Misuse of technology	Domestic violence	5
Cuilco, community 2	*Terapeutas tradicionales*	Misuse of technology	Infidelity	4
Cuilco, community 2	*Terapeutas tradicionales*	Poor physical health	Men's wellbeing	-5
Cuilco, community 2	*Terapeutas tradicionales*	Poor physical health	Emotional distress	4
Cuilco, community 2	*Terapeutas tradicionales*	Substance use	Men's wellbeing	-5
Cuilco, community 2	*Terapeutas tradicionales*	Substance use	Emotional distress	4.67
Cuilco, community 2	*Terapeutas tradicionales*	Substance use	Basic resource insecurity	3
Cuilco, community 2	*Terapeutas tradicionales*	Substance use	Domestic violence	4.5
Cuilco, community 2	*Terapeutas tradicionales*	Substance use	Theft	4.67
Cuilco, community 2	*Terapeutas tradicionales*	Substance use	Misuse of technology	4
Cuilco, community 2	*Terapeutas tradicionales*	Infidelity	Basic resource insecurity	4
Cuilco, community 2	*Terapeutas tradicionales*	Infidelity	Domestic violence	4
Cuilco, community 2	*Terapeutas tradicionales*	Infidelity	Lack of affectionate, trusting, supportive family relationships	3
Cuilco, community 2	*Terapeutas tradicionales*	Infidelity	Poor physical health	5
